# Structural Mechanism of ER Retrieval of MHC Class I by Cowpox

**DOI:** 10.1371/journal.pbio.1001432

**Published:** 2012-11-27

**Authors:** William H. McCoy, Xiaoli Wang, Wayne M. Yokoyama, Ted H. Hansen, Daved H. Fremont

**Affiliations:** 1 Department of Pathology and Immunology, Washington University School of Medicine, St. Louis, Missouri, United States of America; 2 Department of Medicine, Washington University School of Medicine, St. Louis, Missouri, United States of America; 3 Department of Biochemistry and Molecular Biophysics, Washington University School of Medicine, St. Louis, Missouri, United States of America; Whitehead Institute, United States of America

## Abstract

One of the hallmarks of viral immune evasion is the capacity to disrupt major histocompatibility complex class I (MHCI) antigen presentation to evade T-cell detection. Cowpox virus encoded protein CPXV203 blocks MHCI surface expression by exploiting the KDEL-receptor recycling pathway, and here we show that CPXV203 directly binds a wide array of fully assembled MHCI proteins, both classical and non-classical. Further, the stability of CPXV203/MHCI complexes is highly pH dependent, with dramatically increased affinities at the lower pH of the Golgi relative to the endoplasmic reticulum (ER). Crystallographic studies reveal that CPXV203 adopts a beta-sandwich fold similar to poxvirus chemokine binding proteins, and binds the same highly conserved MHCI determinants located under the peptide-binding platform that tapasin, CD8, and natural killer (NK)-receptors engage. Mutagenesis of the CPXV203/MHCI interface identified the importance of two CPXV203 His residues that confer low pH stabilization of the complex and are critical to ER retrieval of MHCI. These studies clarify mechanistically how CPXV203 coordinates with other cowpox proteins to thwart antigen presentation.

## Abstract 2

Structure-function studies reveal how a poxvirus protein blocks cell surface display of MHC class I by exploiting cellular ER retrieval processes.

## Author Summary

Viruses encode a wide array of proteins whose principle function is to disable the surveillance and effector functions of the immune system. A common viral target is the MHC class I antigen processing and presentation pathway, which is a potent mechanism used by the host for the detection and killing of virally infected cells. In this study we have delineated the immune evasion mechanism of the cowpox-encoded CPXV203 protein, which is known to potently block the normal trafficking of MHCI from the endoplasmic reticulum (ER) to the plasma membrane. CPXV203 does this by highjacking an ER-retrieval system that usually serves to capture defective, chaperone complexed MHCI proteins in the Golgi and send them to the ER. We show that CPXV203 adopts a compact beta-sandwich structure and engages evolutionarily conserved MHCI determinants that are located under the peptide-binding platform. The viral protein binds a variety of different MHCI proteins weakly at the pH found in the ER, but the affinity and half-life are significantly augmented at the more acidic conditions found in the Golgi. Together these data suggest that CPXV203 works cooperatively with the endogenous ER retrieval process to promiscuously target fully assembled MHCI, thereby preventing T-cell killing of cowpox infected cells.

## Introduction

Detection of viral infection by CD8 T cells relies on major histocompatibility complex class I (MHCI) presentation of virally derived peptides at the cell surface. Not surprisingly, a wide variety of viruses have evolved mechanisms to disrupt antigen presentation by targeting the assembly and trafficking pathways used by MHCI proteins [1],[2]. The most common immune evasion mechanism appears to be blockade of cytosol-to-endoplasmic reticulum (ER) peptide transport by the transporter associated with antigen processing (TAP) [3]–[10]. However, other viruses target molecular chaperones to impair the quality of peptide loading without curtailing peptide supply [11],[12]. The quality of peptide loading by MHCI is initially controlled by the peptide loading complex (PLC) made up of TAP, tapasin (Tpn), ERp57, and calreticulin (CRT) [13]. Prior to binding an optimal peptide, the PLC retains in the ER nascent MHCI heavy chains (HCs) assembled with beta-2 microglobulin (β2m). Within the PLC, the MHCI-dedicated chaperone Tpn bridges the HC/β2m complex with TAP. Once a peptide of suitable affinity binds to the HC/β2m complex, the fully assembled MHCI is released from the PLC to transit to the cell surface; and perhaps not surprisingly, there are examples of viral immune evasion proteins that impair peptide loading by blocking PLC assembly [11],[12]. In addition to PLC-imposed quality control, non-PLC-associated CRT uses a KDEL-dependent mechanism to retrieve suboptimally loaded MHCI from the early Golgi to the ER to improve peptide binding [14]. This ER retrieval is dependent upon the C-terminal KDEL sequence of CRT that confers binding to the KDEL receptor (KDELR) in the early Golgi in a pH-dependent manner [15].

Several viral immune evasion proteins appear to directly target MHCI proteins, but only adenovirus (AdV) E3-19K and human cytomegalovirus (HCMV) US2 have been shown to directly bind MHCI luminal domains [16],[17]. E3-19K impairs MHCI egress from the ER by either an ER-retention mechanism dependent on its cytoplasmic tail [18] or its ability to prevent Tpn bridging MHCI to TAP [11], while US2 targets MHCI for ER-associated degradation (ERAD) [19]. E3-19K and US2 both exhibit distinct class Ia allele preferences [20]–[22] that may help these viruses evade natural killer (NK) cell cytotoxicity on the basis of missing self [23]. Alternatively, viruses may encode separate proteins to undermine NK cell surveillance [24]. Interestingly, E3-19K has also been reported to target various MHCI assembly intermediates, and mutagenesis analyses suggest that E3-19K may interact with an MHCI surface similar to that bound by US2 [20],[22]. The only structural study of direct MHCI sabotage revealed that US2 uses an Ig-like fold to bind under the MHCI-binding platform near where the N-terminus of the peptide is anchored [25]. Although US2 was crystallized bound to fully assembled MHCI, cellular studies suggest US2 also targets HC before full assembly with peptide and/or β2m [26]. In any case, the structural basis for how US2, E3-19K, or any other viral immune evasion protein discriminates MHCI alleles and/or assembly intermediates has not been previously reported.

While many viruses exhibit strict host specificity, some orthopoxviruses are able to productively infect a wide variety of mammalian species and encode an array of immunomodulatory genes that target both cell intrinsic and extrinsic antiviral responses [27]. Yet until recently, orthopoxviruses were not known to target antigen presentation. The orthopoxvirus cowpox (CPXV) expresses two unrelated immune evasion proteins, CPXV012 and CPXV203 (UniProt [UNP]: Q8QMP2), which use different mechanisms to block antigen presentation in both human and murine cells [28]–[30]. CPXV012 is a small type II transmembrane protein that blocks peptide transport by TAP [29],[30]. By contrast, CPXV203 is a soluble protein that prevents MHCI proteins from trafficking to the plasma membrane by a mechanism dependent upon its C-terminal KTEL sequence, a motif recognized by the KDELR [28]. To initially probe binding partners, Byun et al. (2007) showed that CPXV203 co-precipitated with MHCI and not TAP. These findings implied that CPXV203 binds MHCI lumenal domains or an associated molecule before and/or after peptide assembly [28]. Furthermore, CPXV203 was found to down regulate MHCI proteins in both murine and human cell lines during normal poxvirus infection [29],[30]. This ability to broadly inhibit MHCI by CPXV203 may help explain productive CPXV zoonotic infection of various mammalian species other than small rodents, the apparent CPXV host reservoir [27]. Indeed, mutant cowpox viruses lacking both CPXV012 and CPXV203 demonstrate attenuated virulence in a cytotoxic T lymphocyte (CTL)-dependent manner [29], in contrast to other viral proteins that target MHCI that do not appear to significantly modulate primary infection in vivo [31],[32].

Here we provide a precise understanding of how CPXV203 binds to a broad array of MHCI complexes that includes both classical and non-classical molecules. Biosensor studies indicate that CPXV203 binds MHCI weakly at the pH found in the ER, but the affinity and half-life are significantly augmented at the more acidic conditions found in the Golgi. Crystallographic analysis reveals that CPXV203 adopts a β-sandwich topology reminiscent of poxvirus chemokine-binding proteins, and this domain engages evolutionarily conserved MHCI determinants available only on fully assembled MHCI. We also undertook mutagenesis analysis that supports the structural model and uncovered the critical functional role played by two CPXV203 His residues in the pH regulation of complex stability. Together these data suggest that CPXV203 works cooperatively with the endogenous KDEL-mediated Golgi retrieval process to promiscuously target fully assembled MHCI, thereby preventing T-cell killing of cowpox infected cells.

## Results

### CPXV203 Downregulates Fully Assembled MHCI

**Figure 1:**
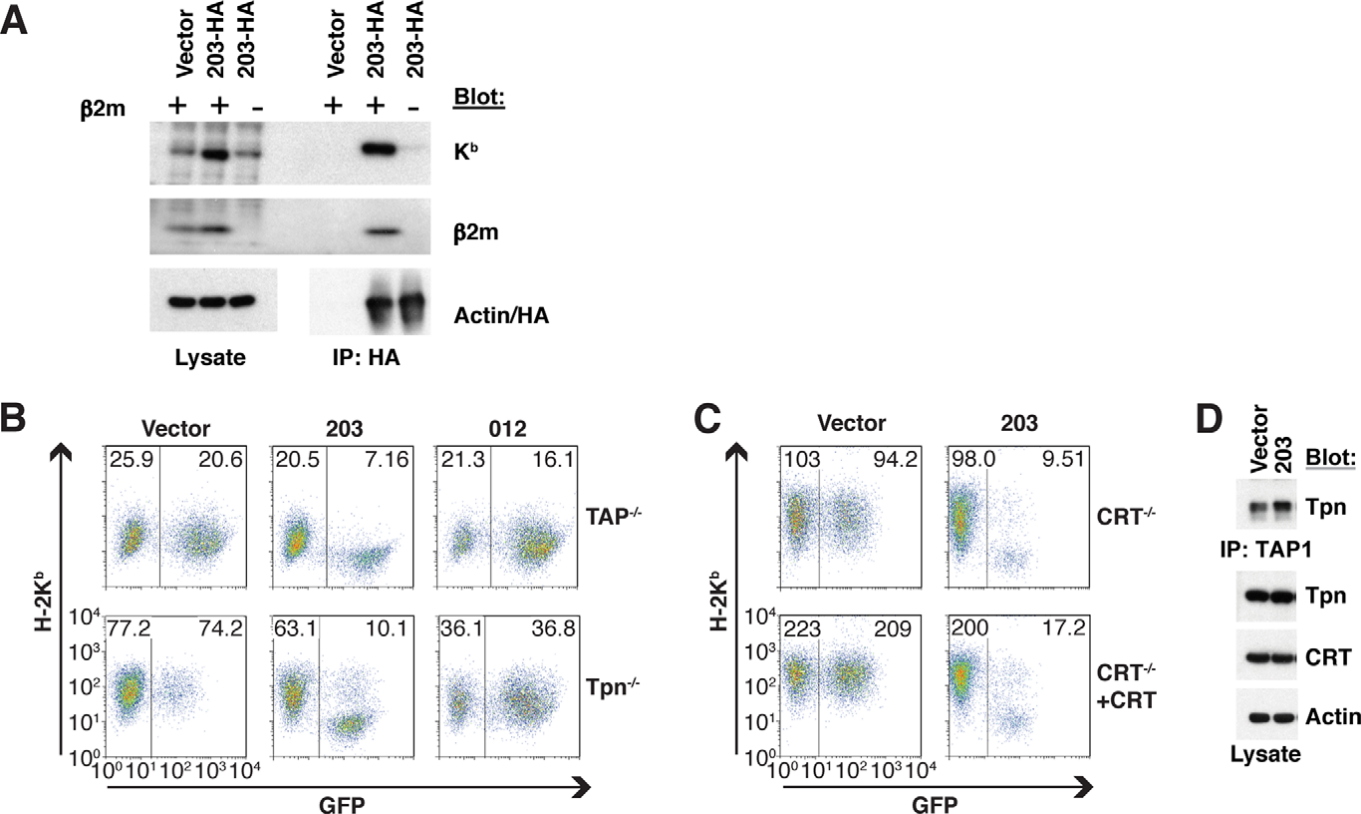
CPXV203 downregulation of MHCI is β2m-dependent but PLC-independent. (A) Hemagglutinin-tagged CPXV203 (CPXV203-HA) stably expressed in β2m^−/−^ cells was unable to co-IP HC. (B) MEFs lacking TAP/Tpn were transduced with CPXV203/CPXV012-IRES-GFP and then MHCI surface expression was monitored by flow-cytometry. B6-derived MEF cell lines expressing TAP/Tpn typically have a mean fluorescence of MHCI surface expression of around 200 as shown in the CRT add-back control in (C), lower left. Data in (B) demonstrate that CPXV203 does not require PLC components to downregulate MHCI, while CPXV012 is TAP/Tpn-dependent. (C) Extension of the studies in B to CRT^−/−^ cells showed that CPXV203 function is independent of CRT. (D) Stable expression of CPXV203 did not impair TAP/Tpn association as monitored by co-IP, and CPXV203 did not reduce steady-state levels of CRT found in cell lysate. Numbers at the top of dot plots in (B) and (C) indicate the mean fluorescence intensity of GFP-negative and -positive populations.

To ascertain which MHCI assembly state(s) is targeted by CPXV203, association with the HC of murine H-2K^b^ (UNP: P01901) was monitored by co-precipitation in wild-type and β2m-deficient cells. CPXV203 only co-precipitated with K^b^ HC in cells expressing β2m (UNP: Q91XJ8) (Figure 1A), suggesting that heterodimer assembly is required for CPXV203/MHCI association. To further assess whether this association was dependent upon the PLC, CPXV203 was expressed by transduction in cells lacking either TAP or Tpn, which present low levels of fully assembled MHCI. As shown in Figure 1B, CPXV203 dramatically reduced MHCI surface expression in cells lacking TAP or Tpn, whereas the TAP inhibitor CPXV012 did not affect surface expression in these PLC-component deficient cells. We also found that CPXV203 comparably downregulates MHCI expression in cells with and without CRT (Figure 1C), suggesting that CPXV203 expression does not grossly disrupt CRT-associated ER quality control as could potentially occur through KDELR competition. In further support of this conclusion, CPXV203 does not interfere with PLC assembly, as shown by normal TAP/Tpn association and normal steady-state levels of CRT (Figure 1D). Previous studies found comparable peptide loading in cells with and without CPXV203, and failed to identify association of CPXV203 with the PLC [28]. Taken together, these findings provide compelling evidence that CPXV203 regulates the surface expression of fully assembled MHCI after dissociation from the PLC without impairing PLC function.

### CPXV203 Engages MHCI in a pH-dependent Manner

**Figure 2:**
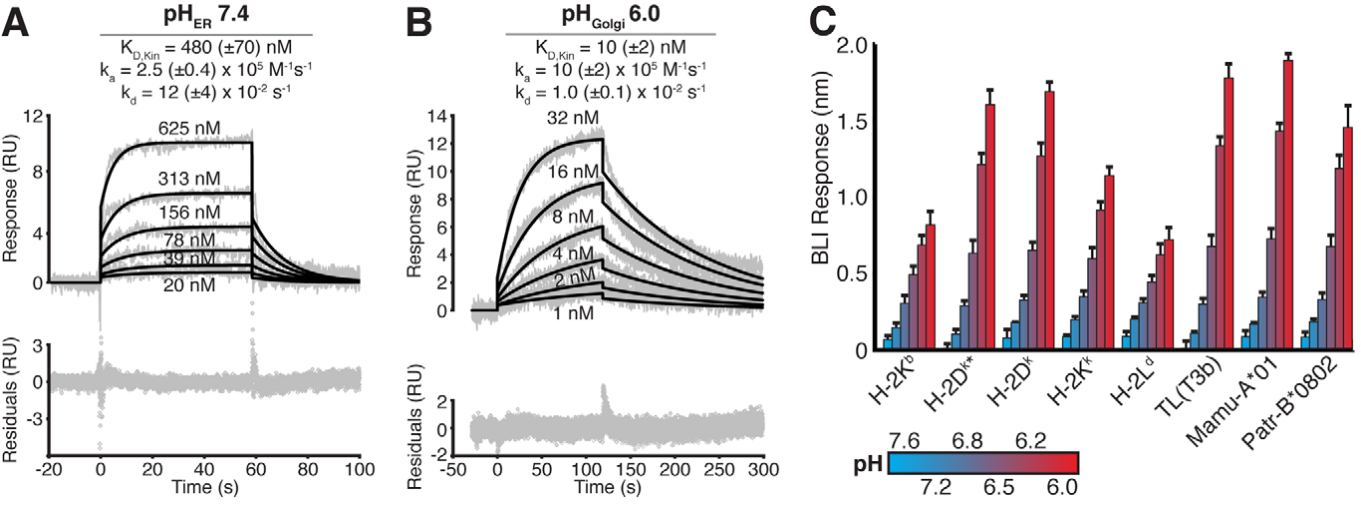
CPXV203 binds MHCI with higher affinity at low pH. (A,B) SPR analysis of CPXV203/K^b^ binding at pH_ER_ 7.4 (A) and pH_Golgi_ 6.0 (B). Neutravidin was used to capture site-specifically biotinylated MHCI prior to injection of increasing concentrations of CPXV203. Non-specific binding was addressed via reference subtraction of a neutravidin-only control flow cell. SPR curves (grey) were fit kinetically using a 1∶1 Langmuir model (black lines). See Table S1 for additional MHCI alleles and Figure S1 for analysis of protein oligomeric state. CPXV203 did not bind β2m alone (pH 6.0/7.4, unpublished data). (C) Equilibrium BLI analysis confirmed CPXV203 affinity increased for murine/primate MHC Ia/Ib alleles as pH decreased from 7.6 to 6.0. Neutravidin sensors captured site-specifically biotinylated MHCI prior to immersion in samples of varying pH with constant [CPXV203]. The [CPXV203] chosen for this experiment produced the lowest detectable signal at pH_ER_ 7.4 for each MHCI. Nonspecific binding was addressed using both reference subtraction (neutravidin) and standard blocking reagents (1% BSA +0.05% TWEEN). *Indicates complex includes murine β2m instead of human β2m.

We next sought to examine whether CPXV203 directly binds to MHCI using soluble recombinant proteins in biophysical assays. We observed that CPXV203 binds K^b^ with an affinity of K_D,Kin_ = 480 nM at pH_ER_ 7.4 using surface-plasmon resonance (SPR) (Figure 2A). The expansion of these studies to additional MHCI molecules revealed that CPXV203 exhibits low affinity interactions (K_D,Kin_ = 82–10,500 nM) with five different murine Ia alleles (D^b^, D^q^, K^b^, K^d^, L^d^) and a primate allele (Ceat-B*12) (Table S1). We also examined a non-classical MHC Ib protein, murine thymic leukemia tumor antigen or TL (T3^b^), which was engaged by CPXV203 with similar affinity and kinetics at pH_ER_ 7.4 as observed for K^b^. Unlike classical MHCI proteins that require peptide loading to assemble, TL pairs with β2m and is stable in the absence of ligand binding. Thus, it appears that the requirement for peptide binding to classical MHC Ia proteins for CPXV203 engagement is based on the role peptide loading plays in assembly and stability rather than direct recognition. Promiscuous CPXV203/MHCI association fits well with the previously published data that CPXV203 downregulates murine H-2D and -K alleles, though the affinities were weaker than those previously reported for the viral ER retention protein E3-19K (11–18 nM) [22].

The weaker than expected affinity of CPXV203 for MHCI led us to evaluate a variety of buffer conditions that might more closely reproduce ER/Golgi conditions (divalent cations: Ca^2+^, Mg^2+^, Zn^2+^; ATP; pH 6–8). Of these changes, only low pH augmented CPXV203/MHCI affinity with a decrease to pH_Golgi_ 6.0 increasing the affinity ∼50-fold (K_D,Kin_ = 10 nM, Figure 2B). This striking enhancement occurs through both an increased on-rate (k_a_) and a decreased off-rate (k_d_) for all tested murine and primate alleles (Table S1). We confirmed these results using a separate biophysical technique, biolayer interferometry (BLI), where the equilibrium response of CPXV203 binding murine (H-2D^k^, -K^b,k^, -L^d^; TL) and primate MHCI (Mamu-A*01, Patr-B*0802) was monitored as a function of pH (7.6–6.0) (Figure 2C). These binding studies demonstrate that the stability of CPXV203/MHCI complexes is pH regulated to favor association in the Golgi rather than the ER. Importantly, similar observations have been made for the binding of KDEL bearing ligands to the KDELR [15].

To address whether enhanced binding of CPXV203 to MHCI at low pH results from changes in the stoichiometry of the complex, multi-angle light scattering (MALS) experiments were undertaken that demonstrated a 1∶1 stoichiometry that was insensitive to pH manipulation from pH 6.5–8.5 (Figure S1). We also undertook circular dichroism spectra analysis that indicated that the conformation of these proteins (alone or in complex) does not change significantly as a function of pH (unpublished data). These results support the 1∶1 binding model used in our biosensor analysis and suggest that CPXV203/MHCI pH regulation likely involves only small local effects.

### Structure of the CPXV203/MHCI Complex

**Figure 3:**
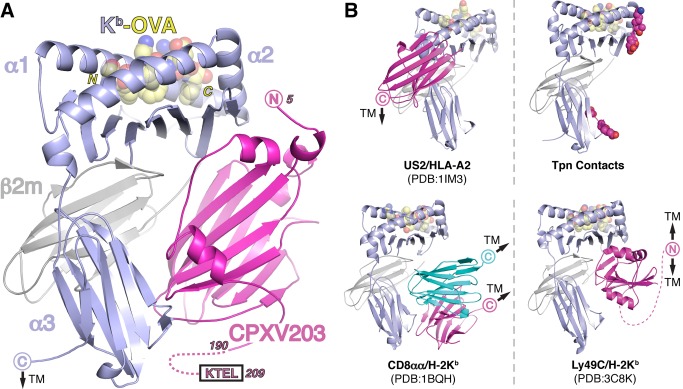
Crystal structure of CPXV203 bound to MHCI. (A) Ribbon diagram of the complex structure of CPXV203 (magenta), K^b^ (blue), β2m (grey), and OVA_257–264_ (yellow, spheres). No N-linked glycosylation sites are present near the interface. Membrane proximal domain shifts, α3 (7.2°) and β2m (16.8°), are within the range observed in previous crystal structures of free MHCI (see Text S1). (B) Comparison of CPXV203/MHCI binding orientation to other MHCI binders: viral (US2) [25], chaperone (Tpn), co-stimulatory (CD8αα) [35], and NK receptor (Ly49C) [40]. Chains colored as in A. Proposed Tpn contact loops (α2 128–136, α3 222–229) are colored magenta with contacts identified by mutagenesis shown as spheres [36],[37],[39],[52]. See also Table S2 and Figure S2.

We next pursued crystallographic studies of CPXV203 in complex with MHCI to better understand the nature of the interaction. Utilizing the observation that CPXV203 binding affinity increases with decreasing pH, we crystallized SeMet-labeled CPXV203 in complex with K^b^ loaded with SIINFEKL (OVA_257–264_) at pH 5.55 and determined the structure at 3.0 Å resolution (Figure 3A; Table S2). Initial molecular replacement phases using MHCI alone were greatly improved through cross-crystal averaging (Figure S2A–S2D) [33], which allowed a preliminary backbone trace of CPXV203 to be built. Subsequently, molecular replacement-single-wavelength anomalous dispersion (MR-SAD) was used to identify eight SeMet sites and introduce anomalous phase information (figure of merit [FOM] 0.604) that improved map quality to the point where the complete CPXV203/MHCI complex could be built and refined. The structure reveals that CPXV203 binds below the MHCI peptide-binding platform, contacting both the HC (α2- and α3-domains) and β2m. Comparison of K^b^ free and bound by CPXV203 did not indicate any significant changes associated with viral protein engagement. The general footprint located below the α2-1 helix of K^b^ is supported by serological experiments whereby we determined whether CPXV203 competed with monoclonal antibodies specific for well-characterized epitopes. Direct binding competition was observed for two monoclonal antibodies (MAbs) (AF6-88.5.3 and Y-3) that have been mapped precisely to this region, and no competition was observed for three MAbs mapped outside of the CPXV203 footprint (Figure S2F–S2H). We note that while HCMV US2 also binds MHCI below the peptide-binding platform, the CPXV203 footprint is completely distinct and, strikingly, overlaps with that of Tpn, CD8, and NK cell receptors (NKRs) (Figure 3B).

### CPXV203 Is Structurally Related to Poxvirus Chemokine-Binding Proteins

**Figure 4:**
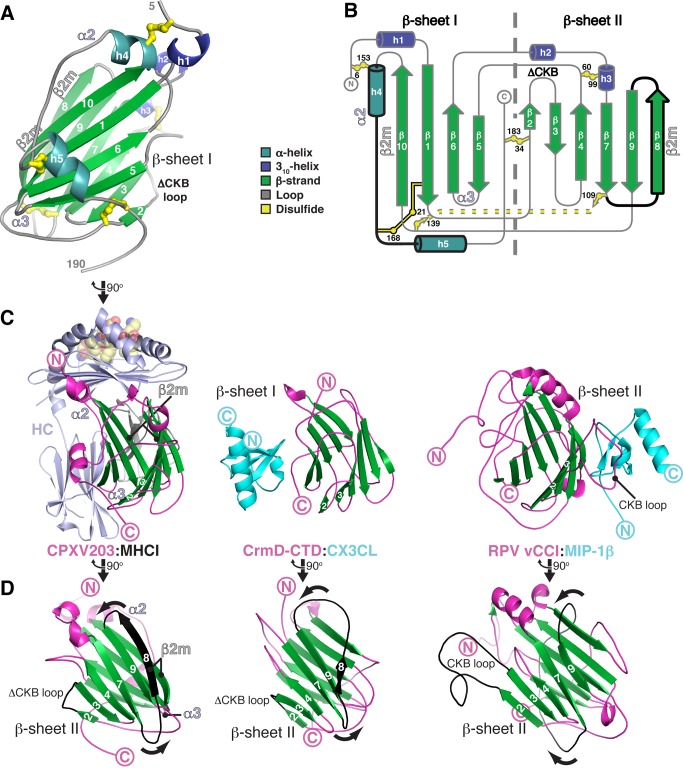
Structural topology of CPXV203 and comparison to poxvirus CKBPs. (A–D) CPXV203 regions used to contact α2, α3, and β2m domains are indicated. (A) Ribbon diagram of CPXV203 (R5-S190) colored according to 2° structure (cyan α helices, blue 3_10_ helices, green β strands, grey loops, and yellow disulfide bonds). CPXV203 orientation is identical to Figure 3A. (B) Topology diagram of CPXV203 with 2° structure coloring as in (A). Disulfide bonds are shown as flattened balls-and-sticks with residue positions listed. The core β sandwich is divided into its β sheets by a dashed, grey line. Structural elements not found in vCCI (PDB: 1CQ3) are highlighted in black, including the absence of a highly negatively charged chemokine-binding (CKB) loop. MHCI α2 and β2m contacts are highly localized to these unique structural elements. (C) CPXV203 and poxvirus CKBPs (RPV vCCI and ECTV CrmD-CTD) use three distinct surfaces for ligand binding. Each CKBP/chemokine (CK) complex (PDBs: 3ONA [53] and 2FFK [54]) was aligned to CPXV203 using CE [55]. The view from Figure 3A has been rotated −90° (y-axis). Complexes are shown as ribbon diagrams: CPXV203 and CKBP (magenta), core β-sandwich (green), H-2K^b^ (blue), β2m (grey), OVA_257–264_ (yellow, spheres), CK (cyan). (D) CPXV203 and CKBPs are shown after a further −90° y-axis rotation to highlight the distinct β7–β9 junction found in CPXV203 and CrmD-CTD relative to vCCI-like proteins. The absence of a CKB loop in CPXV203 and CrmD-CTD relative to vCCI is also indicated. See also Tables S3, S4, and Figure S3.

The structure of CPXV203 does not resemble any structurally characterized viral or host protein known to interact with MHCI. The single domain of CPXV203 (209 aa) is a globular β-sandwich that is stabilized by five disulfide bonds conserved in all T4 poxvirus protein family members (Figures 4A, 4B, S3A). The core β-sandwich consists of two parallel β-sheets (β-sheet I: β1, β5, β6, β10; β-sheet II: β2, β3, β4, β7, β8, β9) made up of anti-parallel strands with one parallel strand interaction (β7/β9) bridging the two segments of β-sheet II (Figure 4B). Three of these disulfide bonds appear to stabilize the h4-loop-h5 arrangement used to engage the MHCI α2-domain. A search for structurally similar proteins indicates that the CPXV203 β-sandwich core resembles the structurally characterized poxvirus chemokine binding proteins (CKBPs), such as the vCCI-like protein encoded by ectromelia virus, EVM001 [34], which exhibits an RMSD of 3.0 Å for 143 aligned residues (Figure S3B; Table S3).

CPXV203 and the poxvirus CKBPs engage their ligands using completely distinct binding surfaces located on opposite faces of the β-sandwich core (Figure 4C). While vCCI-like proteins use β-sheet II to bind chemokines, CPXV203 primarily uses β-sheet I elements. Interestingly, the Ectromelia virus CrmD-CTD (SECRET domain) also appears to use β-sheet I elements to bind chemokines, and it shares with CPXV203 a distinct β7–β9 junction relative to vCCI-like proteins that increases β-sheet I accessibility through the conversion of a flexible loop into a β-sheet II strand (Figure 4D). CPXV203 has further differences with the vCCI core with the replacement of vCCI β13-β14 with two α-helices (h4 and h5), a modification that also exposes CPXV203 β10 to interact with β2m. For CPXV203, these topological changes remove potential steric clashes (Figure S3C), increase solvent-accessibility of the conserved β5–β6 loop (source of nearly all α3 contacts), and create the primary sources for both α2 (h4–h5) and β2m (β8 and β10) contacts (Figure 4B; Table S4). Thus, while CPXV203 is clearly structurally related to poxvirus CKBPs, significant modifications are clearly evident that uniquely allow it to recognize MHCI.

### CPXV203 Contacts Conserved Elements within Each MHCI Domain

**Figure 5:**
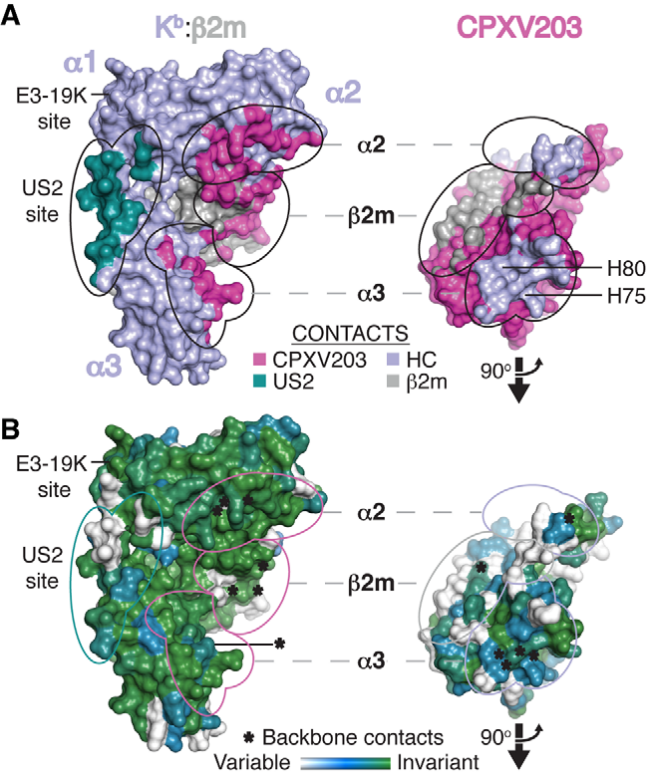
CPXV203 binds conserved elements within each MHCI domain. The CPXV203/MHCI complex is shown as a Connolly surface (1.4 Å probe). Relative to Figure 3A, MHCI was rotated −30° (y-axis), while CPXV203 was rotated 60° (y-axis). Each domain-specific interaction is circled on both molecules. The US2 and E3-19K binding sites are also indicated. (A) CPXV203/MHCI surfaces are colored by chain (see Figure 3A) or by contact (see legend). CPXV203/MHCI interfaces are labeled according to the contacted MHCI domain. Each MHCI domain is contacted by a CPXV203 surface that is localized to a distinct structural region. Comparison of the CPXV203 and US2/E3-19K sites clearly shows the lack of binding site overlap between PLC-proximal and PLC-distal immune evasion proteins. (B) Conservation of interface residues is shown for CPXV203-susceptible MHCI and T4 poxvirus proteins (see Figures S4A and S3A, respectively). Backbone contacts for each interface are indicated (*). CPXV203 contacts highly conserved MHCI surfaces, as opposed to US2 (prominent variable residues between α2–α3). Conservation of MHCI contact residues in the T4 family are highly localized to the α3 interface, which contains 4/6 CPXV203 backbone contact positions. See also Figure S4 and Table S4 and S5.

To understand the structural basis of how CPXV203 interacts with such diverse MHCI-family proteins, we analyzed the conservation of MHCI contacts and the similarity of these contacts to those used by other MHCI-binding proteins. CPXV203 promiscuously binds MHCI through a large, somewhat nonpolar interface divided into three distinct contact regions (α2, α3, and β2m domains) (Figures 5A, 5B, S4A, S4B). The arrangement of these contact regions is only available in fully assembled MHCI, and as such CPXV203 binds MHCI in an assembly-dependent manner that is peptide-independent as long as MHCI assembly is also peptide-independent, as is the case for TL. Comparison of the CPXV203/MHCI interface to similar interfaces reveals that the total buried surface area (BSA) is significantly larger than most other complexes, CPXV203 buries >200 Å^2^ more main-chain (MC) than any similar MHCI-binder, and only CPXV203 divides its interface nearly equally among the platform (α1/α2), β2m, and α3 (Figure 5A; Table S5). CPXV203 recognizes MHCI elements that are extremely well conserved in all murine pMHCI: overall, 86%; CPXV203 contacts, 91%; CPXV203 side chain (SC) contacts, 95%; five invariant SC contacts. Further, CPXV203 recognizes core structural features of the MHCI fold by anchoring each of the three domain interfaces through a buried MC-MC hydrogen bond and two to three MC-SC hydrogen bonds (Figure S4C–S4E; Table S4). Through these contacts, CPXV203 recognizes seven backbone positions conserved by the MHCI fold and coordinates conserved MHCI side chains within the α3 interface (Q226, D227, E229) also required for Tpn and CD8 association [35]–[37]. Further, the presence of CPXV203 His residues opposite negatively charged α3 domain residues (H75-D227, H80-E229) suggests these may be pH-regulated interactions, though only H80-E229 is close enough to form a direct contact (3.5 Å versus 7.3 Å). Finally, we have identified that CPXV203 downregulates the non-classical MHCI molecule H2-M3, while mCD1d escapes CPXV203 retrieval (Figure S4F, S4G). Our structural results support the idea that escape by mCD1d is facilitated in part by a charge reversal at position 229 (mCD1d H233 – CPXV203 H80) and the orientation of mCD1d Q230 away from the interface due to an altered CD-loop conformation (Figure S4H). This structural investigation explains promiscuous MHCI retrieval by CPXV203, as it specifically targets a tri-domain interface of evolutionarily conserved contacts that would only be presented by fully assembled MHCI.

### Critical Role of CPXV203 His-75 and His-80 in pH-Regulated MHCI Interactions

**Figure 6:**
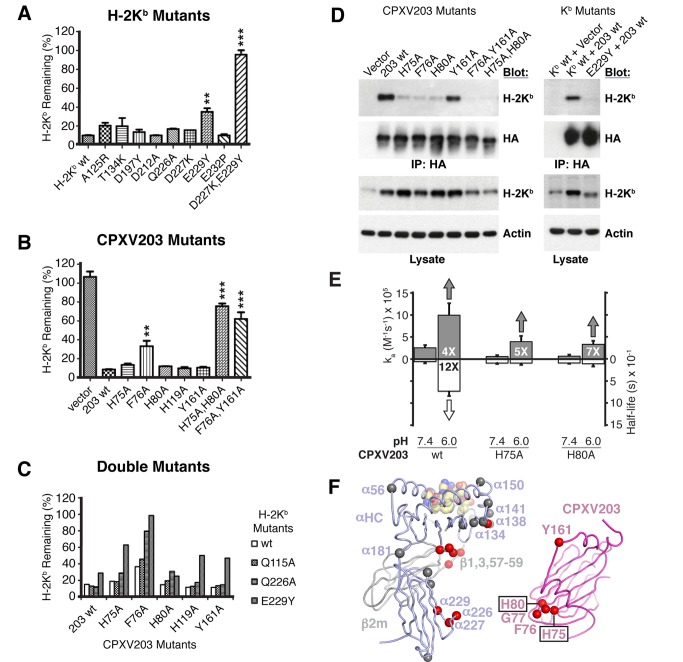
Mutagenesis supports a tri-domain interface and pH regulation. (A–C) 3KO cells stably transduced with β2m and K^b^ (3KO-β2m-K^b^) were transduced with CPXV203-IRES-GFP to evaluate the effect of CPXV203/MHCI interface mutation on surface K^b^ remaining relative to untransduced cells. Comparable expression of wild-type and mutant proteins was determined using co-expressed GFP and/or western blotting. Each figure represents the data from two independent experiments. **p<0.01; ***p<0.001 when compared with wild type. Limited MHCI escape was observed for two single α3 interface mutations (K^b^ E229Y or CPXV203 F76A), while significant K^b^ escape required double mutation of the α3 interface (K^b^ D227K/E229Y, CPXV203 H75A/H80A, K^b^ Q226A/CPXV203 F76A, K^b^ E229Y/CPXV203 H75A, K^b^ E229Y/CPXV203 F76A) or simultaneous mutation of interfaces α2 and α3 (CPXV203 F76A, Y161A) (D) Most mutant CPXV203-HA exhibited a decreased ability to co-IP K^b^ even though surface K^b^ (%) did not increase significantly. CPXV203/K^b^ mutations that significantly increased surface K^b^ expression also exhibited a decrease in total K^b^ (lysate), indicative of the release of MHCI retention [28]. (E) Kinetic analysis (SPR) of alanine mutation of α3 interface histidines (CPXV203 H75 and H80) summarized by on-rate (k_a_, (+)-axis) and half-life (half-life, (−)-axis). (F) Summary of mutagenic analysis (functional and biosensor) mapped to CPXV203/MHCI structure (same colors as Figure 3A) shown as cartoon loops except for the peptide (spheres). Mutated residues (Cα spheres) were colored to indicate whether they had a significant effect (red) or no significant effect (grey). CPXV203 and MHCI have been translated apart (x-axis) to highlight corresponding interface mutations. See also Tables S6, S7.

We assessed the functional relevance of specific determinants within the CPXV203/MHCI binding interface by extensive mutagenesis of both CPXV203 and K^b^. Mutants were assayed for loss of function by rescue of surface K^b^ expression or lack of physical association by co-immunoprecipitation (co-IP). Single mutations in either K^b^ or CPXV203 from all three interaction sites (Figure 5) were tested, but only α3 interface mutations K^b^ E229Y and CPXV203 F76A significantly rescued K^b^ surface expression (Figure 6A, 6B). Furthermore, double mutations within the α3 interface (K^b^ D227K/E229Y, CPXV203 H75A/H80A, K^b^ Q226A/CPXV203 F76A, K^b^ E229Y/CPXV203 H75A, K^b^ E229Y/CPXV203 F76A) or the simultaneous mutation of interfaces α2 and α3 (CPXV203 Y161A, F76A) significantly enhanced K^b^ rescue, with some mutants displaying complete ablation of CPXV203 function (Figure 6A–6C). Physical association (CPXV203-HA/K^b^) was impaired more dramatically than K^b^ rescue by single α3 interface mutations (Figure 6D), though it should be noted that the HA-tag might impair association. In any case, these experiments clearly demonstrate the functional importance of our structurally defined interface in CPXV203-mediated MHCI association and retrieval.

To extend these findings, biosensor studies were undertaken to probe the contribution of individual interface residues in binding and pH regulation. Equilibrium analysis (BLI, pH_ER_ 7.4) of CPXV203 and MHCI mutants further confirmed the three-site binding footprint (Figure 5) and clearly distinguished the CPXV203 binding site from those of E3-19K, US2, and MAb Y-3 (Table S6). Alanine mutation of several CPXV203 residues within the α3-domain interface (including His residues 75 and 80) had a pronounced deficit in binding (similar to co-IP). CPXV203 H75 and H80 were selected for kinetic analysis (SPR) based on their chemical environment (Figure S4E) and previously described sensitivity to alanine mutation (functional and association). Alanine mutation of either His ablated the off-rate (k_d_) enhancement at low pH, while maintaining a similar on-rate (k_a_) enhancement (Figure 6E; Table S7). Thus at low pH, α3 interface His residues act to extend the CPXV203/MHCI half-life (6 s–73 s), while a separate interaction appears to regulate the faster association (k_a_) observed at low pH. Consistent with their importance, the double mutant (H75A, H80A) displayed extremely weak affinity that prohibited accurate kinetic analysis, though equilibrium BLI assays clearly support the greater functional deficit observed for this mutant (Table S6). These investigations indicate that CPXV203 engages MHCI through critical pH-regulated interactions with conserved MHCI α3-domain determinants, while the α2 and β2m domain interfaces may enable CPXV203 to bind fully assembled MHCI with broad specificity.

## Discussion

**Figure 7:**
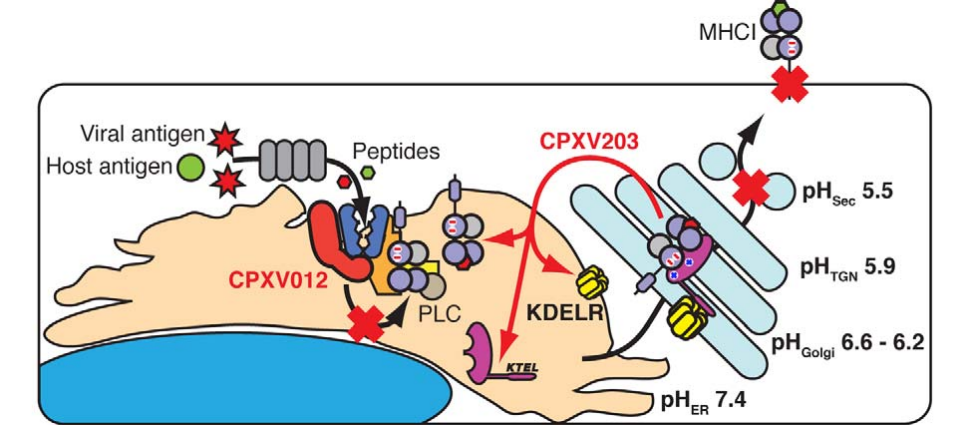
CPXV203 co-opts the KDELR to retrieve MHCI from the Golgi. Comparison of the separate but complementary strategies employed by CPXV012 and CPXV203 to downregulate MHCI. CPXV012 impairs nascent MHCI folding by inhibiting TAP, while CPXV203 selectively binds mature MHCI in the low pH environment found in the ERGIC-Golgi and then uses the KDELR to return to the ER where KDELR/CPXV203/MHCI can rapidly dissociate.

Viral infection of mammalian hosts can be greatly facilitated by viral proteins that confer the ability to evade CTL detection and clearance. Not surprisingly, viruses have evolved a wide variety of strategies to reduce cell surface presentation of viral peptides on MHCI [1],[2]. The cellular, structural, and biophysical results reported here provide a complete picture of one such strategy, as CPXV203 was shown to directly bind fully assembled MHCI in a manner that is regulated via the normal pH gradient that exists between the ER and Golgi compartments (Figure 7). Though CPXV203 makes contacts to three distinct MHCI domains, pH regulation of the complex half-life is critically dependent on CPXV203 His residues that bind to an α3-domain acidic CD loop important for both Tpn and CD8 association. Thus, CPXV203 exploits a cellular pathway to target MHCI surfaces critical for immunological function in a manner that selects for those MHCI molecules most likely to present viral peptides.

Remarkably, CPX203 is not related to any other MHCI-binding protein, but rather it is most structurally related to poxvirus CKBPs. To our knowledge, CPXV203 is the first member of the large T4 poxvirus protein family [38] to be structurally characterized, suggesting a previously underappreciated link between the poxvirus CKBPs and T4 protein families through similarities in their β-sandwich core. Regardless of the evolutionary history, the adaptation of this protein domain to structurally distinct ligands and unrelated functional outcomes suggests the integral role that CPXV203 plays in antigen presentation disruption may not be its only function.

CPXV203 evolved into a promiscuous MHCI-binding protein by targeting MHCI determinants that are largely conserved by virtue of their roles in the recognition by host factors essential to cellular immunity (Tpn, CD8, NKRs). For instance, many α2-domain contacts (R111, Q115, E128, T134) are conserved through Tpn (α2 128–136) [37],[39] and Ly49 (R111, Q115, D122) [40] interactions, while the β2m contacts primarily involve structurally conserved backbone positions within the LIR-1/MHCI interface [41]. The direct overlap of CD8 and Tpn contact sites (Q226, D227, E229) [35]–[37] in the acidic CD loop of the α3 domain is clearly exploited by CPXV203 for MHCI binding, and this interface is precisely where we have identified two His residues in the viral protein that regulate increased kinetic stability at the lower pH of the Golgi.

Previous investigations of pH-dependent endosomal (PRL/PRLr [42], FcRn/Fc [43]) and ER→Golgi (RAP/LRP) [44] trafficking have repeatedly identified His residues as the pH sensitive component of these regulatory mechanisms. Unlike other amino acids, histidine is well suited to serve this function, as small pH shifts can drastically change the charge and hydrogen-bonding potential of this residue. As such, our investigation of CPXV203/MHCI pH regulation focused on interface histidines, which revealed a significant contribution of CPXV203 H75 and H80 to complex half-life at low pH. We suggest that these titratable His residues endow CPXV203 with the ability to regulate fully assembled MHCI in a manner that is complementary to the regulation of PLC-associated MHCI by CPXV012.

The specific binding of CPXV203 to fully assembled MHCI proteins in a pH-dependent manner clarifies mechanistically how CPXV203 coordinates with CPXV012 to effectively block antigen presentation. Previous characterizations showed the CPXV012 functions in a PLC-dependent fashion to block TAP transport of peptide into the ER [29],[30]. However, some MHCI-binding peptides in the ER are not TAP-dependent and the CPXV012 block of peptide transport is likely not absolute. The MHCI proteins that are able to bind peptide in the presence of CPXV012 are left to CPXV203, since it binds fully assembled MHCI through domain-specific conformational determinants conserved in classical and many non-classical MHCI. Among these interactions, the α3 interface is particularly important based on the presence of CPXV203 His residues that impart pH regulation to the CPXV203/MHCI interaction. This pH dependence suggests that CPXV203/MHCI interacts most avidly in the Golgi and not the ER, thus limiting the pool of MHCI that CPXV203 must retrieve.

Interestingly, CRT has a C-terminal KDEL sequence conferring ER retrieval, and non-PLC-associated CRT has recently been implicated in quality control of MHCI peptide loading [14]. More specifically, CRT was shown to accumulate in the *cis*-Golgi and return peptide accessible MHCI proteins to the ER. Both CRT and CPXV203 retrieve MHCI proteins but with opposite goals. CRT functions in host quality control by retrieving MHCI with suboptimal peptides, whereas CPXV203 functions in immune evasion by retrieving fully assembled MHCI to block antigen presentation. Thus CPXV012 and -203 act sequentially to efficiently block MHCI expression using PLC-dependent versus PLC-independent mechanisms, respectively. As a possible consequence of efficient MHCI downregulation resulting in NK cell susceptibility, CPXV expresses the soluble class I-like protein OMCP that functions as a competitive antagonist of the NKG2D-activating receptor [24]. Indeed, the combined sabotage of both CTL and NK cell detection of virus-infected cells explains why mutant CPXV lacking CPXV012 and 203 demonstrates attenuated virulence in vivo compared to wild-type virus [29].

## Methods

### Antibodies

MHCI-specific MAbs used in SPR competition assays were obtained from the ATCC (H-2K^b^: 25-D1.1.6, B8-24-3, Y-3), purchased from BioLegend (K^b^: AF6-88.5.3, K^b^/D^b^: 28-8-6, K^d^/D^d^: 34-1-2S, hβ2m: 2M2), or provided as a kind gift (K^b^: 5F1-2-14) from S. Nathenson (Albert Einstein College of Medicine, New York) and L. Pease (Mayo Clinic, Minnesota). MAbs that were not purchased from BioLegend were purified from ascites on a Bio-Rad Profinia FPLC using Protein A or G. MAbs used in flow-cytometry and IP assays have been described previously. MAb footprints in Figure S2H are based on SPR data from this work and available literature (Text S1).

### Peptides

Peptides were synthesized by Fmoc chemistry and then subjected to reverse-phase HPLC for purification. Peptides were resuspended at >1 mM in ddH_2_O, DMSO, or 6M GuHCl, as dictated by peptide solubility. Peptides were chosen based on available MHCI crystal structures or personal suggestions by A. Stout (NIH Tetramer Core Facility). See Text S1 for a list of all peptides used in this study.

### Cell Lines

Murine embryo fibroblast (MEF) B6/WT3 (WT3) and mutant MEFs including TAP1-deficient cells (FT1^−^), Tapasin-deficient cells (Tpn^−/−^), calreticulin-deficient cells (CRT^−/−^), β2m-deficient cells (B6.B2M^−^) and triple knockout fibroblasts (K^b−/−^ D^b−/−^ β2m^−/−^; 3KO) were all derived from C57BL/6 (H-2b) embryos and have been described previously [45]. The CPXV203 and K^b^ mutants were stably expressed in the indicated cells by retroviral expression vectors pMXsIG [28] and pMIN [45], respectively. Cells transduced by pMIN were selected by neomycin while green fluorescent protein (GFP^+^) cells from pMXsIG transduced lines were enriched by cell sorting.

### Immunoprecipitations, Immunoblot, and Cross-linking

For co-IPs (TAP1/Tpn and CPXV203-HA/H-2K^b^), cells were lysed in PBS with 1.0% digitonin (Wako) and protease inhibitor cocktails (Roche) for 60 min. Post-nuclear lysates were then incubated with indicated antibodies + protein A-sepharose (Sigma) or anti-HA sepharose (sigma) for HA-tagged CPXV203 for 1 h. After washes, coprecipitated proteins were eluted by boiling in lithium dodecyl sulfate (LDS) sample buffer (Invitrogen). For cross-linking treatment (CPXV203-HA/H-2K^b^), cells were incubated with 1–2 mM DSP (Thermo) in PBS for 2 h at 4°C. The cross-linking was terminated with 25 mM Tris-HCl pH 7.4 before the cells were lysed in PBS with 1.0% NP-40. Following immunoprecipitation cross-linked proteins were eluted by boiling in LDS sample butter with 2.5% β-mercaptoethanol. Immunoblot of precipitated proteins was performed following SDS-PAGE separation. Specific proteins were visualized by chemiluminescence using the ECL system (Thermo).

### Flow Cytometry

All flow cytometric analyses were performed using a FACS Calibur (Becton Dickinson). Data were analyzed using FlowJo software (Tree Star). Staining was performed as described [46]. PE-conjugated goat anti-mouse IgG (BD Pharmingen) was used to visualize MHCI staining. PE-conjugated anti-mouse CD1d (eBioscience) was used to detect surface CD1d. GFP signal representing CPXV203 transduced cells were collected in the FITC channel.

### Protein Production

Mammalian CPXV203ΔKTEL (aa 1–205, etgMVI-LHV), bacterial CPXV203ΔKTEL (aa 1–205, maMVI-LHV), and bacterial MHCI were produced using established methods (Text S1). K^b^ (aa 0–280, mGPH-PST)/H-2K^d^ (aa 0–283, mGPH-VSN) constructs were produced in house, while H-2D^d^, H-2D^k^, H-2D^q^, H-2K^k^, H-2L^d^, Mamu-A*01, Patr-B*0802, H-2Q9-H-2D^b^, H-2K^b^-HLA-A*0201, HLA-A*0201-H-2K^b^ were produced by the NIH Tetramer Core Facility. H-2D^b^ biotinylated monomer (LCMV Gp33, KAVYNFATC) was purchased from Beckman Coulter. Biotinylated constructs included a C-terminal site-specific biotinylation tag and were biotinylated by following established procedures (Avidity). Chimeric mCD1d-Fc produced in a murine cell line was obtained from R&D Systems. All MHCI include human β2m (hβ2m, aa 0–99, mIQR-RDM, UNP: P61769) unless otherwise noted. Signal-peptide/cloning artifacts are indicated as lower-case aa.

### X-ray Structure Determination

CPXV203 (Brighton Red strain, SeMet-labeled)/MHCI(OVA_257–264_:H-2K^b^:hβ2m) complex was prepared for crystallization by size-exclusion chromatography purification of CPXV203/MHCI in low pH/salt buffer (50 mM NaCl, 30 mM MES pH 5.6, 0.01% Azide). Diffraction-quality crystals of CPXV203/MHCI were grown at 20°C by streak seeding into hanging drops of 0.5 µl complex (7.5 mg/ml) +0.5 µl reservoir solution (10% PEG 6000, 4% glucose, 2% ethylene glycol, 0.1 M tri-K citrate pH 5.55, 0.01% Azide). Crystals were dehydrated, flash frozen in liquid N_2_, and then used for X-ray data collection at the Advanced Light Source (ALS) beamline 4.2.2 (0.97909 Å wavelength). Crystals belong to space group P1 (a = 88.31 Å, b = 88.25 Å, c = 106.42 Å; α = 76.18°, β = 69.29°, γ = 66.69°) with four CPXV203/MHCI complexes per asymmetric unit (ASU) and a solvent content of 52%. The HKL2000 software package [47] was used to index, integrate, and scale the data, yielding an 87.9% complete dataset at 3.0 Å (R-sym = 16.4%) (Table S2). The structure of H-2K^bm8^:mβ2m (2CLZ) [48] was used for molecular replacement (MR) using Phaser within the Phenix suite [49], with four MHCI proteins located within the ASU. Electron density for the unique CPXV203 region was improved by cross-crystal averaging [33] allowing an initial model of CPXV203 to be manually built in Coot [50]. MR-SAD using AutoSol (Phenix suite [49]) was subsequently used to identify eight SeMet sites in the four CPXV203 monomers, enabling the introduction of anomalous phase information (figure of merit [FOM] 0.604) that improved map quality allowing for complete CPXV203/MHCI model building. Phenix Refine [49] was used with global non-crystallographic symmetry (NCS) restraints to refine the CPXV203/MHCI structure to a final R_work_ of 22.9% and R_free_ 25.3% (see Table S2 for complete crystallographic statistics). The final CPXV203/MHCI model contains four CPXV203/MHCI complexes and 72 water molecules. Each complex contains the nearly complete CPXV203 (mature residues 5–190), K^b^ (mature residue 1–277), human β2m (0M-99), and OVA_257–264_. All structure figures were created using PyMOL [51]. See Text S1 for comprehensive crystallization, data collection, structure determination, refinement, structural analysis, and figure preparation details.

### Biophysical Characterization of CPXV203/MHCI Interaction

SPR experiments were run on a Biacore T100 (GE Healthcare) in either standard HBS-EP+ (pH 7.4) or low pH MBS-EP+ (pH 6.0). Kinetic and equilibrium analyses were performed using Biacore T100 Evaluation software using a 1∶1 Langmuir model. BLI experiments were performed on an Octet RED system (ForteBio) using HBS-EP+/MBS-EP+ supplemented with 0.05% (v/v) TWEEN and 1% BSA. Equilibrium BLI data was analyzed using Octet software (V7.0). All biosensor experiments were run at 25°C and followed proper biosensor experimental technique. Size-exclusion chromatography-multi-angle light scattering (SEC-MALS) experiments were run on a Dawn HELEOS-II 18-angle light scattering detector (Wyatt) and Optilab rEX refractive index monitor (Wyatt) linked to a Waters HPLC system. Dynamic light scattering (DLS) was performed on a DynaPro-801TC. Circular dichroism was measured using a Jasco-810 instrument (Jasco Inc.). Detailed methodologies for biosensor, light scattering, and circular dichroism experiments are available in Text S1.

## Supporting Information

Figure S1 **Oligomeric state of CPXV203, MHCI, and complex as a function of pH.** (A,B) MALS was run on a DAWN HELEOS system. All samples were applied at 1 mg/ml (15–42 µM) and underwent SEC (20°C) on a WTC-030S5 column prior to entering the DAWN system. (A) CPXV203/MHCI is a 1∶1 complex at physiologic pH 7.4. These proteins alone and in complex behave as single, well-behaved species with MALS MW that matches the calculated MW. Complex analysis utilized a high-affinity MHCI mutant (Y84A, C121S; see Table S6) that did not dissociate during the course of SEC, as wild-type complex dissociated during SEC at pH_ER_ 7.4. MALS analysis of both wt and mutant MHCI produced MW_MALS_ that matched MW_Calc_ for a MHCI heterotrimer. Mass spectrometry determined MW for CPXV203 is also listed. (B) MALS evaluation of these proteins from pH 8.5–6.5 indicates oligomeric state (alone or in complex) remains constant across pH 8.5–6.5. Complex analysis in this experiment again utilized the high-affinity MHCI mutant (Y84A, C121S), which also exhibits pH-enhanced binding at low pH (unpublished data). MALS analysis of wild-type complex (unpublished data) showed a reduction in complex dissociation as pH decreased, which correlates with the increased affinity observed in biosensor experiments. Dynamic light scattering (DLS) experiments run on a DynaPro-801TC supports stable 1∶1 binding stoichiometry and stable oligomeric state from pH 7.5–5.4 for CPXV203 and wt MHCI (unpublished data).
(TIF)

Figure S2 **CPXV203 electron density improvement and validation.** (A–C) Electron density (2σ) in the CPXV203 region is shown using phases from molecular replacement (A), cross-crystal averaging (B), or the final model (C). MHCI is displayed as in Figure 3A. The Cα trace of CPXV203 is shown in (C). Though the contiguous density available for model building was significantly improved by non-crystallographic symmetry (NCS), successful model building required the improvement in estimated phases from cross-crystal averaging against a large number of MHCI complexes and free MHCI. (D) The cross-crystal averaged electron density (1σ) for part of CPXV203 β6 (black box) is shown to illustrate the electron density that was available to initiate CPXV203 model building using a stretch of residues with bulky side chains (H80, Y82, E84, F85). SA-OMIT and 2Fo-Fc maps are shown for comparison. All electron density images were produced in PyMOL using isomesh at the designated σ. (E) Stereo image of the CPXV203 backbone trace is shown as sticks colored from N- to C-terminus using a spectrum from blue to red, respectively. Relative to Figure 3A, CPXV203 was rotated 60° (y-axis) to optimize the view of the MHCI binding surface. Note the structural elements unique to CPXV203 that are involved in MHCI binding: β5–β6 hairpin loop (green), β8 (yellow), β10 (orange), h4–h5 (red). (F–H) Serological studies localized CPXV203/MHCI interface below the α2-1 helix. (F) An SPR adaption of a sandwich ELISA was used to evaluate the interface. In brief, MAbs immobilized through amine coupling were used to capture MHCI prior to an injection of tetramerized CPXV203. An increased RU signal during the injection would indicate that the MAb did not block the CPXV203/MHCI interface, while free dissociation of MHCI would indicate the MAb blocked the interface (directly or sterically). Examples of both outcomes are shown. (G) MHCI from our structural work is shown as described in Figure 3A with labels identifying MHCI domains and the α2-1 helix. (H) The mAb footprints are shown as shaded circles on MHCI. Green indicates non-blocking antibodies, while red indicates blocking antibodies. MAb footprints are based on published cellular and structural studies (see Text S1). The AF6-88.5.3 footprint was refined in this work through the observation that this MAb does not recognize mutant H-2K^b^ (M228T) and that it competes with Y-3.
(TIF)

Figure S3 **CPXV203 alignments and modeling.** (A,B) Alignments of CPXV203 indicate residue conservation colored by similarity (ACILMPV DE FHWY HKR NQST), experimentally observed 2° structure (CPXV203, above; CPXV vCCI, below), side chain accessibility (CPXV203, above; CPXV vCCI, below), and MHCI contacts. CPXV203 histidines are marked with an asterisk, except for pH-regulated interface histidines (see Figure 6) that are labeled with blue/red circles. CPXV203 E162 is labeled with a blue/grey circle to indicate it is used in both the α2 and β2m interfaces. Side chain solvent accessibility was defined using NACCESS (1.4 Å probe); black circles, <30% accessible; grey circles, 30%–60% accessible; white circles, >60% accessible. (A) The sequence of CPXV203 was aligned to T4 poxvirus proteins with a broad range of sequence identity; taterapox (TATV, 96%), monkeypox (MPXV, 64%), yokapox (YPV, 60%), deerpox (DPV, 34%), and myxoma (MYXV, 26%). Virus strains are indicated in parentheses. The C-terminal α-helix predicted by all 2° structure prediction programs used in these studies is shown in white. Cysteine residues (ten conserved positions) are boxed in yellow, while the conserved C-terminal KDEL-variant is boxed in red. Predicted N-linked glycosylation sites are boxed in green. As CPXV203 N146 is not solvent-accessible, its lack of glycosylation (mass spec and PNGase treatment, unpublished data) is not surprising. (B) Structure-based alignment (Dali server) of CPXV203 with various poxvirus CKBPs: ectromelia virus (ECTV) CrmD C-terminal domain (CrmD-CTD) [1], vaccinia virus (VACV) A41 [2], ECTV vCCI [3], rabbitpox (RPV) vCCI [4], and cowpox virus (CPXV) vCCI [5]. Contact residues identified through structural studies (CPXV203, CrmD-CTD, RPV vCCI) or mutagenesis (ECTV vCCI) are shown above (MHCI) or below (CK) the alignments. PDB IDs for proteins in this alignment: 3ON9, 2VGA, 2GRK, 2FFK, 1CQ3. (C) Poxvirus CKBPs vCCI and CrmD-CTD were modeled (CE alignment of CKBP to CPXV203 [6]) onto the CPXV203/MHCI complex to evaluate potential steric clashes due to structural elements found in CKBPs (blue) but absent in CPXV203. The complexes are shown using the same representation, color scheme, and orientation as in Figure 3A. All topological differences between CKBPs and CPXV203 are listed. Connolly surfaces are shown for severe clashes (≤2 Å).
(TIF)

Figure S4 **CPXV203/MHCI interface analysis.** (A,B) Sequence alignment of MHCI regions contacted by CPXV203. MHCI 2^o^ structure, Tpn/CD8/NKR contacts, and side chain solvent accessibility are shown above the alignments (see Figure S3 for additional alignment details). CPXV203/MHCI contacts are boxed in magenta, while MHCI backbone contacts are indicated with an asterisk above the alignment. (A) MHCI HC were included in this alignment if they are downregulated or bound by CPXV203. Murine CD1d was identified as a null allele in this study (see G), and so it is shown below the other alleles to highlight the lack of sequence conservation in CPXV203/MHCI contact regions. D227 and E229 are indicated with blue/red circles, as CPXV203 H75 and H80 are believed to contact these Tpn contact residues in a pH-regulated manner. (B) β2m sequences of potential poxvirus hosts are shown (primate, rodent, livestock). Tpn contacts are based on published mutagenesis work [7]–[10], while HBPLUS was used to identify CD8 and NKR contacts from all available complex structures (CD8/MHCI: 1AKJ, 1BQH, 1NEZ, 3DMM; Ly49/MHCI: 1QO3, 3C8K; LIR/MHCI: 1P7Q, 2DYP) [11]–[19]. (C–E) The CPXV203/MHCI complex is shown using stick representation with each interface colored by chain (as in Figure 3A) and element (C, blue/grey/magenta; O, red; N, dark blue). Side-chains are shown for all contact residues, and MHCI backbone contacts are indicated (*). Residues that appear to be involved in putative hydrogen-bond networks (interface contacts: yellow dots; potential β5–β6 loop conformation stabilizers: grey dots) are labeled. Interface views were rotated (relative to Figure 3A) for optimal viewing: C 10° (y); D 90° (y); E ∼90° (x), ∼150° (z). (C) CPXV203 contacts the underside of the MHCI peptide-binding platform (α1/α2) through burial of large hydrophobic residues (I160 and Y161) immediately following helix-5 (h5). (D) Parallel CPXV203 histidines from β-sheets I and II coordinate β2m backbone carbonyl oxygens. The β2m interface MC-MC hydrogen bond is above the shown view. (E) CPXV203 extensively interacts with conserved MHCI residues Q226 and E229. CPXV203 H75 and H80 are directly opposite conserved, acidic MHCI residues D227 and E229. (F,G) CPXV203 is able to downregulate MHC Ia and Ib molecules but not mCD1d. WT3 cells were transduced with either vector or CPXV203-IRES-GFP and then MHCI surface expression was monitored by flow-cytometry. (F) For H2-M3 expression, cells were incubated with 20 µM Fr38 (fFMIVIL) peptide from *Listeria monocytogenes* at 37°C over night (18 h) before staining by MAb 130, as previously described [20]. CPXV203 downregulated both the Ia (H-2K^b^) and Ib (H2-M3) molecules. Similar studies revealed that H-2K^k^ (Ia) and MR1 (Ib) are both susceptible to CPXV203-mediated downregulation (data not shown). (G) Extension of the studies to L929-CD1d cells (H-2k) revealed that CPXV203 was not able to downregulate the Ib molecule CD1d, while the Ia molecule H-2D^k^ was efficiently downregulated. SPR and BLI studies at pH_ER_ and pH_Golgi_ also confirmed mCD1d as a null allele (unpublished data). (H) Comparison of MHCI crystal structures clearly shows conservation of the α3 loop conformation with the exception of CD1d. The α3 domains (181–274) of numerous MHCI structures (murine and human, Ia and Ib) were aligned by combinatorial extension. A subset of those structures is shown here (PDBs 1VAC, 1P7Q, 3ILP) [18],[21],[22].
(TIF)

Table S1 **SPR analysis of CPXV203/MHCI binding.**
(DOCX)

Table S2 **Summary of data collection, phasing, and refinement.**
(DOCX)

Table S3 **Structural comparison of CPXV203 with vCCI-like CKBPs.**
(DOCX)

Table S4 **CPXV203/MHCI interactions.**
(DOCX)

Table S5 **Buried surface area comparison of CPXV203/MHCI to similar interfaces.**
(DOCX)

Table S6 **BLI analysis of mutant CPXV203/MHCI binding.**
(DOCX)

Table S7 **SPR analysis of mutant CPXV203/MHCI binding.**
(DOCX)

Text S1 **Supplemental methods.**
(DOCX)

## Abbreviations

β2m: beta-2 microglobulin
BLI: biolayer interferometry
CK: chemokine
CKB: chemokine binding
CKBP: chemokine binding protein
co-IP: co-immunoprecipitation
CPXV: cowpox virus
CRT: calreticulin
CTL: cytotoxic T lymphocyte
ER: endoplasmic reticulum
GFP: green fluorescent protein
HC: heavy chain
k_a_: on-rate
k_d_: off-rate
KDELR: KDEL receptor
MAb: monoclonal antibody
MALS: multi-angle light scattering
MC: main-chain
MEF: murine embryo fibroblast
MHCI: major histocompatibility complex class I
NK: natural killer
NKR: natural killer cell receptor
PLC: peptide loading complex
SPR: surface-plasmon resonance
TAP: transporter associated with antigen processing
TL: thymic leukemia tumor antigen, Tpn, tapasin
UNP: UniProt

## References

[1] Hansen TH, Bouvier M (2009) MHC class I antigen presentation: learning from viral evasion strategies. Nat Rev Immunol 9: 503–513. 19498380 10.1038/nri2575

[2] Lilley BN, Ploegh HL (2005) Viral modulation of antigen presentation: manipulation of cellular targets in the ER and beyond. Immunol Rev 207: 126–144. 16181332 10.1111/j.0105-2896.2005.00318.x

[3] Früh K, Ahn K, Djaballah H, Sempé P, van Endert PM, et al (1995) A viral inhibitor of peptide transporters for antigen presentation. Nature 375: 415–418. 7760936 10.1038/375415a0

[4] Hill A, Jugovic P, York I, Russ G, Bennink J, et al (1995) Herpes simplex virus turns off the TAP to evade host immunity. Nature 375: 411–415. 7760935 10.1038/375411a0

[5] Ahn K, Gruhler A, Galocha B, Jones TR, Wiertz EJ, et al (1997) The ER-luminal domain of the HCMV glycoprotein US6 inhibits peptide translocation by TAP. Immunity 6: 613–621. 9175839 10.1016/s1074-7613(00)80349-0

[6] Hengel H, Koopmann JO, Flohr T, Muranyi W, Goulmy E, et al (1997) A viral ER-resident glycoprotein inactivates the MHC-encoded peptide transporter. Immunity 6: 623–632. 9175840 10.1016/s1074-7613(00)80350-7

[7] Lehner PJ, Karttunen JT, Wilkinson GW, Cresswell P (1997) The human cytomegalovirus US6 glycoprotein inhibits transporter associated with antigen processing-dependent peptide translocation. Proc Natl Acad Sci U S A 94: 6904–6909. 9192664 10.1073/pnas.94.13.6904 PMC21257

[8] Koppers-Lalic D, Verweij MC, Lipińska AD, Wang Y, Quinten E, et al (2008) Varicellovirus UL 49.5 proteins differentially affect the function of the transporter associated with antigen processing, TAP. PLoS Pathog 4: e1000080 doi:10.1371/journal.ppat.1000080. 18516302 10.1371/journal.ppat.1000080 PMC2386557

[9] Hislop AD, Ressing ME, van Leeuwen D, Pudney VA, Horst D, et al (2007) A CD8+ T cell immune evasion protein specific to Epstein-Barr virus and its close relatives in Old World primates. J Exp Med 204: 1863–1873. 17620360 10.1084/jem.20070256 PMC2118677

[10] Horst D, van Leeuwen D, Croft NP, Garstka MA, Hislop AD, et al (2009) Specific targeting of the EBV lytic phase protein BNLF2a to the transporter associated with antigen processing results in impairment of HLA class I-restricted antigen presentation. J Immunol 182: 2313–2324. 19201886 10.4049/jimmunol.0803218

[11] Bennett EM, Bennink JR, Yewdell JW, Brodsky FM (1999) Cutting edge: adenovirus E19 has two mechanisms for affecting class I MHC expression. J Immunol 162: 5049–5052. 10227971

[12] Park B, Kim Y, Shin J, Lee S, Cho K, et al (2004) Human cytomegalovirus inhibits tapasin-dependent peptide loading and optimization of the MHC class I peptide cargo for immune evasion. Immunity 20: 71–85. 14738766 10.1016/s1074-7613(03)00355-8

[13] Peaper DR, Cresswell P (2008) Regulation of MHC class I assembly and peptide binding. Annu Rev Cell Dev Biol 24: 343–368. 18729726 10.1146/annurev.cellbio.24.110707.175347

[14] Howe C, Garstka M, Al-Balushi M, Ghanem E, Antoniou AN, et al (2009) Calreticulin-dependent recycling in the early secretory pathway mediates optimal peptide loading of MHC class I molecules. EMBO J 28: 3730–3744. 19851281 10.1038/emboj.2009.296 PMC2790484

[15] Capitani M, Sallese M (2009) The KDEL receptor: new functions for an old protein. FEBS Lett 583: 3863–3871. 19854180 10.1016/j.febslet.2009.10.053

[16] Kämpe O, Bellgrau D, Hammerling U, Lind P, Pääbo S, et al (1983) Complex formation of class I transplantation antigens and a viral glycoprotein. J Biol Chem 258: 10594–10598. 6885796

[17] Gewurz BE, Wang EW, Tortorella D, Schust DJ, Ploegh HL (2001) Human cytomegalovirus US2 endoplasmic reticulum-lumenal domain dictates association with major histocompatibility complex class I in a locus-specific manner. J Virol 75: 5197–5204. 11333901 10.1128/JVI.75.11.5197-5204.2001 PMC114925

[18] Pääbo S, Bhat BM, Wold WS, Peterson PA (1987) A short sequence in the COOH-terminus makes an adenovirus membrane glycoprotein a resident of the endoplasmic reticulum. Cell 50: 311–317. 2954653 10.1016/0092-8674(87)90226-1 PMC7133293

[19] Wiertz EJ, Tortorella D, Bogyo M, Yu J, Mothes W, et al (1996) Sec61-mediated transfer of a membrane protein from the endoplasmic reticulum to the proteasome for destruction. Nature 384: 432–438. 8945469 10.1038/384432a0

[20] Burgert HG, Kvist S (1987) The E3/19K protein of adenovirus type 2 binds to the domains of histocompatibility antigens required for CTL recognition. EMBO J 6: 2019–2026. 2958275 10.1002/j.1460-2075.1987.tb02466.x PMC553591

[21] Machold RP, Wiertz EJHJ, Jones TR, Ploegh HL (1997) The HCMV gene products US11 and US2 differ in their ability to attack allelic forms of murine major histocompatibility complex (MHC) class I heavy chains. J Exp Med 185: 363–366. 9016885 10.1084/jem.185.2.363 PMC2211711

[22] Liu H, Fu J, Bouvier M (2007) Allele- and locus-specific recognition of class I MHC molecules by the immunomodulatory E3-19K protein from adenovirus. J Immunol 178: 4567–4575. 17372015 10.4049/jimmunol.178.7.4567

[23] Yokoyama WM, Plougastel BFM (2003) Immune functions encoded by the natural killer gene complex. Nat Rev Immunol 3: 304–316. 12669021 10.1038/nri1055

[24] Campbell JA, Trossman DS, Yokoyama WM, Carayannopoulos LN (2007) Zoonotic orthopoxviruses encode a high-affinity antagonist of NKG2D. J Exp Med 204: 1311–1317. 17548517 10.1084/jem.20062026 PMC2118624

[25] Gewurz BE, Gaudet R, Tortorella D, Wang EW, Ploegh HL, et al (2001) Antigen presentation subverted: Structure of the human cytomegalovirus protein US2 bound to the class I molecule HLA-A2. Proc Natl Acad Sci U S A 98: 6794–6799. 11391001 10.1073/pnas.121172898 PMC34432

[26] Loureiro J, Lilley BN, Spooner E, Noriega V, Tortorella D, et al (2006) Signal peptide peptidase is required for dislocation from the endoplasmic reticulum. Nature 441: 894–897. 16738546 10.1038/nature04830

[27] McFadden G (2005) Poxvirus tropism. Nat Rev Microbiol 3: 201–213. 15738948 10.1038/nrmicro1099 PMC4382915

[28] Byun M, Wang X, Pak M, Hansen TH, Yokoyama WM (2007) Cowpox virus exploits the endoplasmic reticulum retention pathway to inhibit MHC class I transport to the cell surface. Cell Host Microbe 2: 306–315. 18005752 10.1016/j.chom.2007.09.002

[29] Byun M, Verweij MC, Pickup DJ, Wiertz EJHJ, Hansen TH, et al (2009) Two mechanistically distinct immune evasion proteins of cowpox virus combine to avoid antiviral CD8 T cells. Cell Host Microbe 6: 422–432. 19917497 10.1016/j.chom.2009.09.012 PMC2791900

[30] Alzhanova D, Edwards DM, Hammarlund E, Scholz IG, Horst D, et al (2009) Cowpox virus inhibits the transporter associated with antigen processing to evade T cell recognition. Cell Host Microbe 6: 433–445. 19917498 10.1016/j.chom.2009.09.013 PMC2791678

[31] Gold MC, Munks MW, Wagner M, McMahon CW, Kelly A, et al (2004) Murine cytomegalovirus interference with antigen presentation has little effect on the size or the effector memory phenotype of the CD8 T cell response. J Immunol 172: 6944–6953. 15153514 10.4049/jimmunol.172.11.6944

[32] Hansen SG, Powers CJ, Richards R, Ventura AB, Ford JC, et al (2010) Evasion of CD8+ T Cells Is Critical for Superinfection by Cytomegalovirus. Science 328: 102–106. 20360110 10.1126/science.1185350 PMC2883175

[33] Li W, Li F (2011) Cross-crystal averaging with search models to improve molecular replacement phases. Structure 19: 155–161. 21300285 10.1016/j.str.2010.12.007 PMC3037595

[34] Arnold PL, Fremont DH (2006) Structural determinants of chemokine binding by an Ectromelia virus-encoded decoy receptor. J Virol 80: 7439–7449. 16840324 10.1128/JVI.00576-06 PMC1563704

[35] Kern PS, Teng MK, Smolyar A, Liu JH, Liu J, et al (1998) Structural basis of CD8 coreceptor function revealed by crystallographic analysis of a murine CD8alphaalpha ectodomain fragment in complex with H-2Kb. Immunity 9: 519–530. 9806638 10.1016/s1074-7613(00)80635-4

[36] Carreno BM, Solheim JC, Harris M, Stroynowski I, Connolly JM, et al (1995) TAP associates with a unique class I conformation, whereas calnexin associates with multiple class I forms in mouse and man. J Immunol 155: 4726–4733. 7594473

[37] Yu YY, Turnquist HR, Myers NB, Balendiran GK, Hansen TH, et al (1999) An extensive region of an MHC class I alpha 2 domain loop influences interaction with the assembly complex. J Immunol 163: 4427–4433. 10510384

[38] Barry M, Hnatiuk S, Mossman K, Lee SF, Boshkov L, et al (1997) The myxoma virus M-T4 gene encodes a novel RDEL-containing protein that is retained within the endoplasmic reticulum and is important for the productive infection of lymphocytes. Virology 239: 360–377. 9434727 10.1006/viro.1997.8894

[39] Lewis JW, Elliott T (1998) Evidence for successive peptide binding and quality control stages during MHC class I assembly. Curr Biol 8: 717–720. 9637925 10.1016/s0960-9822(98)70280-5

[40] Deng L, Cho S, Malchiodi EL, Kerzic MC, Dam J, et al (2008) Molecular architecture of the major histocompatibility complex class I-binding site of Ly49 natural killer cell receptors. J Biol Chem 283: 16840–16849. 18426793 10.1074/jbc.M801526200 PMC2423261

[41] Willcox BE, Thomas LM, Bjorkman PJ (2003) Crystal structure of HLA-A2 bound to LIR-1, a host and viral major histocompatibility complex receptor. Nat Immunol 4: 913–919. 12897781 10.1038/ni961

[42] Kulkarni MV, Tettamanzi MC, Murphy JW, Keeler C, Myszka DG, et al (2010) Two independent histidines, one in human prolactin and one in its receptor, are critical for pH-dependent receptor recognition and activation. J Biol Chem 285: 38524–38533. 20889499 10.1074/jbc.M110.172072 PMC2992285

[43] Martin WL, West AP Jr, Gan L, Bjorkman PJ (2001) Crystal structure at 2.8 A of an FcRn/heterodimeric Fc complex: mechanism of pH-dependent binding. Mol Cell 7: 867–877. 11336709 10.1016/s1097-2765(01)00230-1

[44] Lee D, Walsh JD, Mikhailenko I, Yu P, Migliorini M, et al (2006) RAP uses a histidine switch to regulate its interaction with LRP in the ER and Golgi. Mol Cell 22: 423–430. 16678114 10.1016/j.molcel.2006.04.011

[45] Lybarger L, Wang X, Harris MR, Virgin HW 4th, Hansen TH (2003) Virus subversion of the MHC class I peptide-loading complex. Immunity 18: 121–130. 12530981 10.1016/s1074-7613(02)00509-5

[46] Yu YYL, Harris MR, Lybarger L, Kimpler LA, Myers NB, et al (2002) Physical association of the K3 protein of gamma-2 herpesvirus 68 with major histocompatibility complex class I molecules with impaired peptide and beta(2)-microglobulin assembly. J Virol 76: 2796–2803. 11861847 10.1128/JVI.76.6.2796-2803.2002 PMC135993

[47] Otwinowski Z, Minor W (1997) [20] Processing of X-ray diffraction data collected in oscillation mode. Macromolecular crystallography part A, volume 276. Waltham (Massachusetts): Academic Press. pp. 307–326. Available: http://www.sciencedirect.com/science/article/pii/S007668799776066X. Accessed 30 December 2011. 10.1016/S0076-6879(97)76066-X 27754618

[48] Auphan-Anezin N, Mazza C, Guimezanes A, Barrett-Wilt GA, Montero-Julian F, et al (2006) Distinct orientation of the alloreactive monoclonal CD8 T cell activation program by three different peptide/MHC complexes. Eur J Immunol 36: 1856–1866. 16761314 10.1002/eji.200635895

[49] Adams PD, Afonine PV, Bunkóczi G, Chen VB, Echols N, et al. (2011) The Phenix software for automated determination of macromolecular structures. Methods (San Diego). Available: http://www.ncbi.nlm.nih.gov/pubmed/21821126. Accessed 30 September 2011. 10.1016/j.ymeth.2011.07.005 PMC3193589 21821126

[50] Emsley P, Cowtan K (2004) Coot: model-building tools for molecular graphics. Acta Crystallogr D Biol Crystallogr 60: 2126–2132. 15572765 10.1107/S0907444904019158

[51] The PyMOL Molecular Graphics System (n.d.). New York: Schrödinger, LLC.

[52] Suh WK, Derby MA, Cohen-Doyle MF, Schoenhals GJ, Früh K, et al (1999) Interaction of murine MHC class I molecules with tapasin and TAP enhances peptide loading and involves the heavy chain alpha3 domain. J Immunol 162: 1530–1540. 9973410

[53] Xue X, Lu Q, Wei H, Wang D, Chen D, et al (2011) Structural basis of chemokine sequestration by CrmD, a poxvirus-encoded tumor necrosis factor receptor. PLoS Pathog 7: e1002162 doi:10.1371/journal.ppat.1002162. 21829356 10.1371/journal.ppat.1002162 PMC3145792

[54] Zhang L, Derider M, McCornack MA, Jao S-C, Isern N, et al (2006) Solution structure of the complex between poxvirus-encoded CC chemokine inhibitor vCCI and human MIP-1beta. Proc Natl Acad Sci U S A 103: 13985–13990. 16963564 10.1073/pnas.0602142103 PMC1599900

[55] Shindyalov IN, Bourne PE (1998) Protein structure alignment by incremental combinatorial extension (CE) of the optimal path. Protein Eng 11: 739–747. 9796821 10.1093/protein/11.9.739

