# Supplementary figures and images for: Structural Mechanism of ER Retrieval of MHC Class I by Cowpox
Source: PLoS Biol. 2012 Nov 27;10(11):e1001432. doi: 10.1371/journal.pbio.1001432 (PMC3507924; doi:10.1371/journal.pbio.1001432)

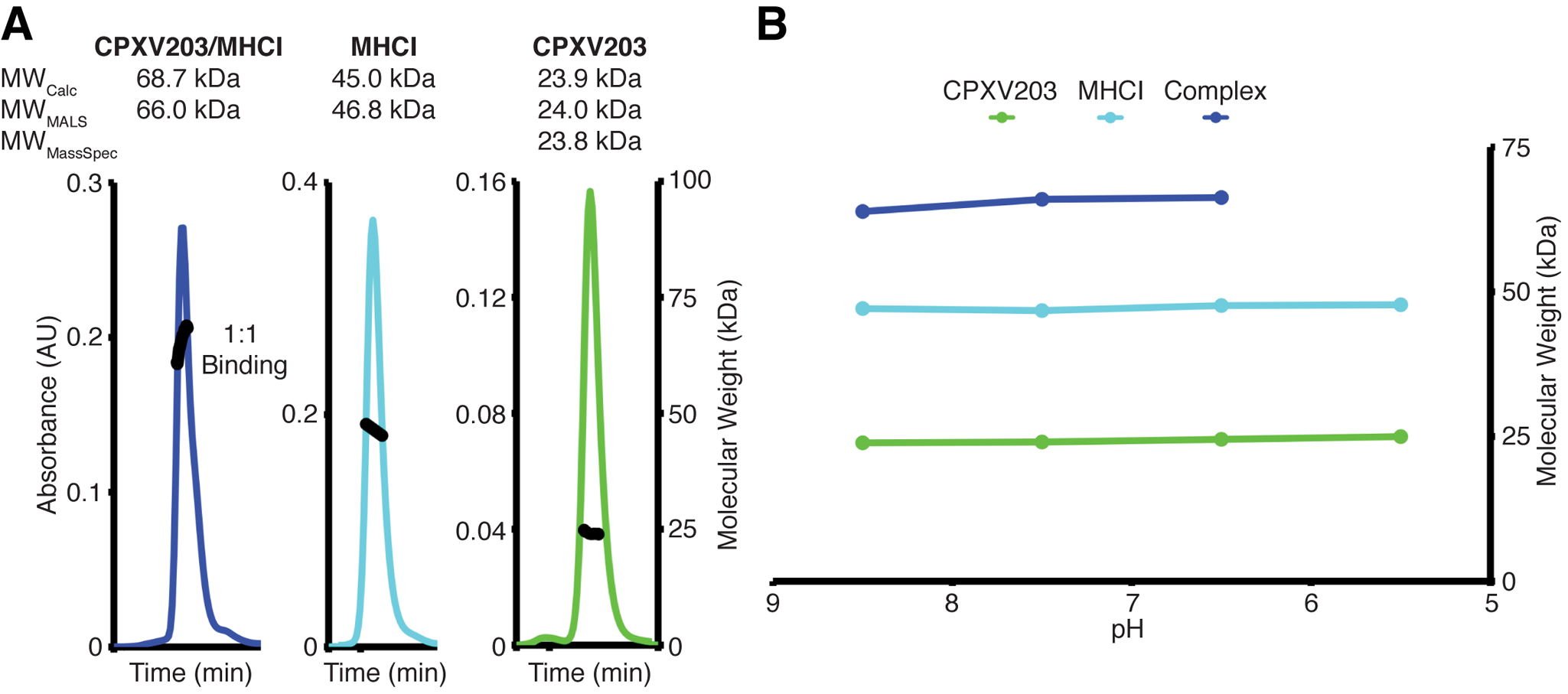

Supplement: Figure S1 — Oligomeric state of CPXV203, MHCI, and complex as a function of pH. (A,B) MALS was run on a DAWN HELEOS system. All samples were applied at 1 mg/ml (15–42 µM) and underwent SEC (20°C) on a WTC-030S5 column prior to entering the DAWN system. (A) CPXV203/MHCI is a 1∶1 complex at physiologic pH 7.4. These proteins alone and in complex behave as single, well-behaved species with MALS MW that matches the calculated MW. Complex analysis utilized a high-affinity MHCI mutant (Y84A, C121S; see Table S6) that did not dissociate during the course of SEC, as wild-type complex dissociated during SEC at pHER 7.4. MALS analysis of both wt and mutant MHCI produced MWMALS that matched MWCalc for a MHCI heterotrimer. Mass spectrometry determined MW for CPXV203 is also listed. (B) MALS evaluation of these proteins from pH 8.5–6.5 indicates oligomeric state (alone or in complex) remains constant across pH 8.5–6.5. Complex analysis in this experiment again utilized the high-affinity MHCI mutant (Y84A, C121S), which also exhibits pH-enhanced binding at low pH (unpublished data). MALS analysis of wild-type complex (unpublished data) showed a reduction in complex dissociation as pH decreased, which correlates with the increased affinity observed in biosensor experiments. Dynamic light scattering (DLS) experiments run on a DynaPro-801TC supports stable 1∶1 binding stoichiometry and stable oligomeric state from pH 7.5–5.4 for CPXV203 and wt MHCI (unpublished data). (TIF) [file pbio.1001432.s001.tif]

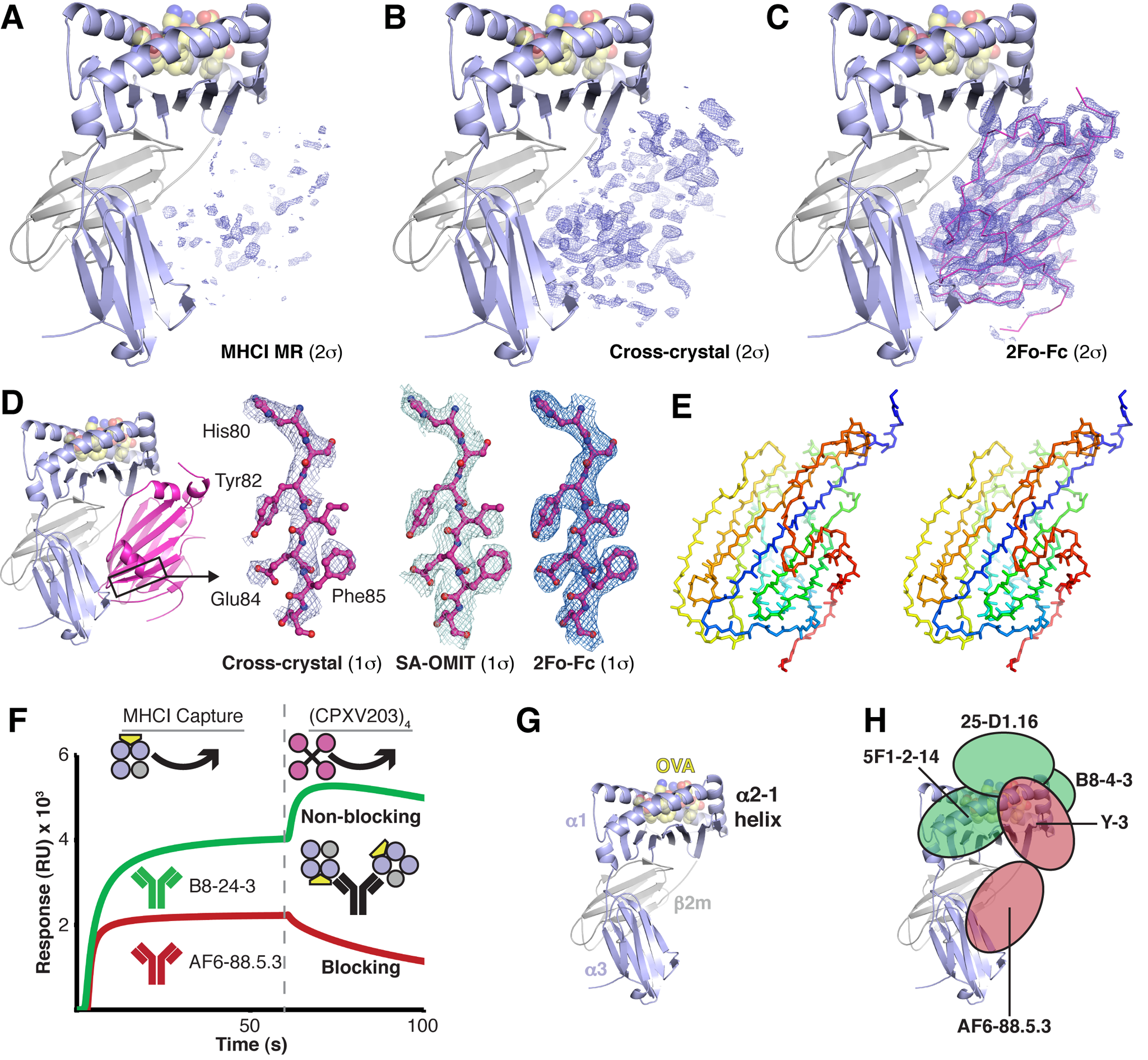

Supplement: Figure S2 — CPXV203 electron density improvement and validation. (A–C) Electron density (2σ) in the CPXV203 region is shown using phases from molecular replacement (A), cross-crystal averaging (B), or the final model (C). MHCI is displayed as in Figure 3A. The Cα trace of CPXV203 is shown in (C). Though the contiguous density available for model building was significantly improved by non-crystallographic symmetry (NCS), successful model building required the improvement in estimated phases from cross-crystal averaging against a large number of MHCI complexes and free MHCI. (D) The cross-crystal averaged electron density (1σ) for part of CPXV203 β6 (black box) is shown to illustrate the electron density that was available to initiate CPXV203 model building using a stretch of residues with bulky side chains (H80, Y82, E84, F85). SA-OMIT and 2Fo-Fc maps are shown for comparison. All electron density images were produced in PyMOL using isomesh at the designated σ. (E) Stereo image of the CPXV203 backbone trace is shown as sticks colored from N- to C-terminus using a spectrum from blue to red, respectively. Relative to Figure 3A, CPXV203 was rotated 60° (y-axis) to optimize the view of the MHCI binding surface. Note the structural elements unique to CPXV203 that are involved in MHCI binding: β5–β6 hairpin loop (green), β8 (yellow), β10 (orange), h4–h5 (red). (F–H) Serological studies localized CPXV203/MHCI interface below the α2-1 helix. (F) An SPR adaption of a sandwich ELISA was used to evaluate the interface. In brief, MAbs immobilized through amine coupling were used to capture MHCI prior to an injection of tetramerized CPXV203. An increased RU signal during the injection would indicate that the MAb did not block the CPXV203/MHCI interface, while free dissociation of MHCI would indicate the MAb blocked the interface (directly or sterically). Examples of both outcomes are shown. (G) MHCI from our structural work is shown as described in Figure 3A with labels identifying MHCI doma [file pbio.1001432.s002.tif]

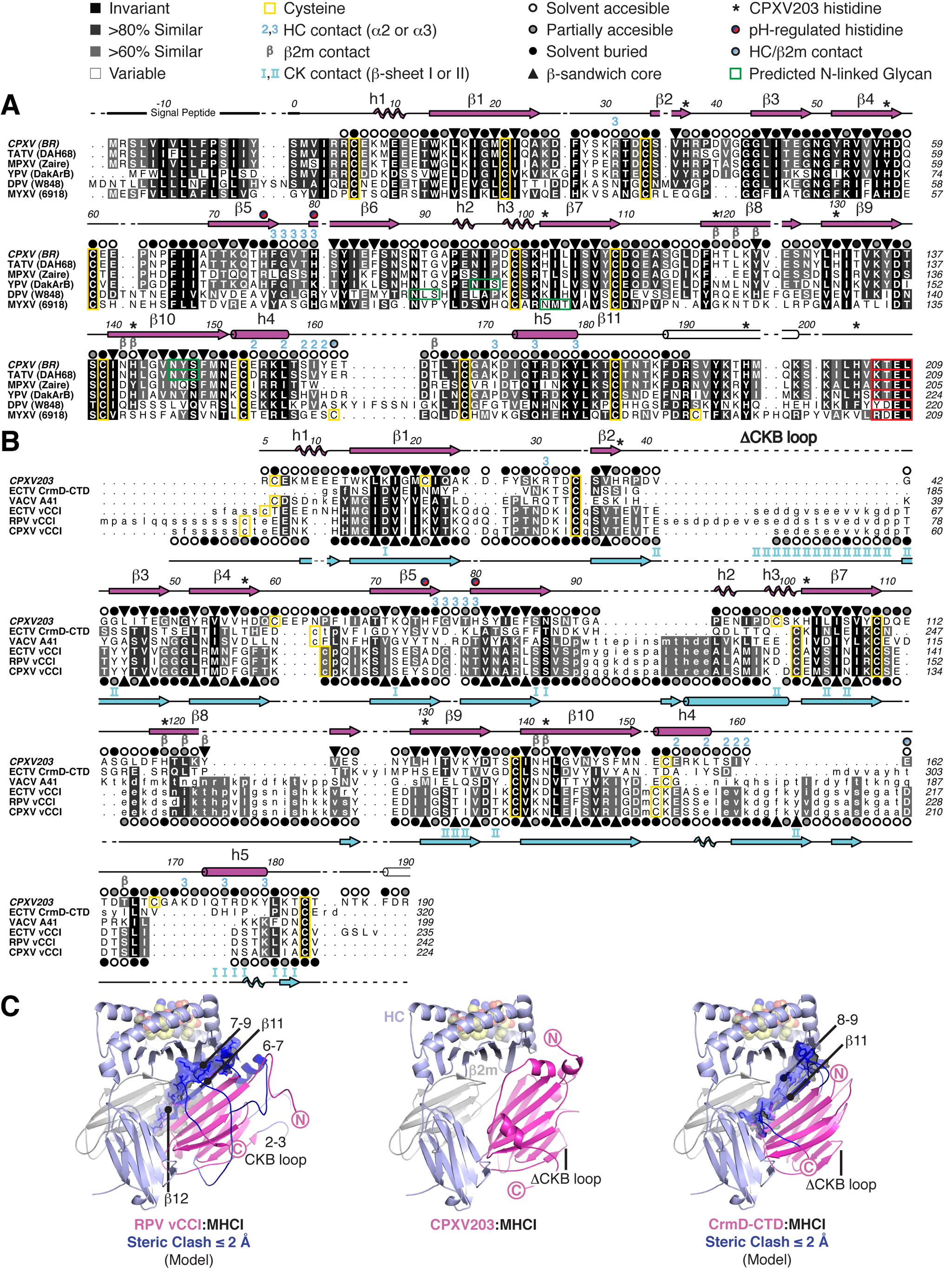

Supplement: Figure S3 — CPXV203 alignments and modeling. (A,B) Alignments of CPXV203 indicate residue conservation colored by similarity (ACILMPV DE FHWY HKR NQST), experimentally observed 2° structure (CPXV203, above; CPXV vCCI, below), side chain accessibility (CPXV203, above; CPXV vCCI, below), and MHCI contacts. CPXV203 histidines are marked with an asterisk, except for pH-regulated interface histidines (see Figure 6) that are labeled with blue/red circles. CPXV203 E162 is labeled with a blue/grey circle to indicate it is used in both the α2 and β2m interfaces. Side chain solvent accessibility was defined using NACCESS (1.4 Å probe); black circles, <30% accessible; grey circles, 30%–60% accessible; white circles, >60% accessible. (A) The sequence of CPXV203 was aligned to T4 poxvirus proteins with a broad range of sequence identity; taterapox (TATV, 96%), monkeypox (MPXV, 64%), yokapox (YPV, 60%), deerpox (DPV, 34%), and myxoma (MYXV, 26%). Virus strains are indicated in parentheses. The C-terminal α-helix predicted by all 2° structure prediction programs used in these studies is shown in white. Cysteine residues (ten conserved positions) are boxed in yellow, while the conserved C-terminal KDEL-variant is boxed in red. Predicted N-linked glycosylation sites are boxed in green. As CPXV203 N146 is not solvent-accessible, its lack of glycosylation (mass spec and PNGase treatment, unpublished data) is not surprising. (B) Structure-based alignment (Dali server) of CPXV203 with various poxvirus CKBPs: ectromelia virus (ECTV) CrmD C-terminal domain (CrmD-CTD) [1], vaccinia virus (VACV) A41 [2], ECTV vCCI [3], rabbitpox (RPV) vCCI [4], and cowpox virus (CPXV) vCCI [5]. Contact residues identified through structural studies (CPXV203, CrmD-CTD, RPV vCCI) or mutagenesis (ECTV vCCI) are shown above (MHCI) or below (CK) the alignments. PDB IDs for proteins in this alignment: 3ON9, 2VGA, 2GRK, 2FFK, 1CQ3. (C) Poxvirus CKBPs vCCI and CrmD-CTD were modeled (CE alignment of CKBP to CPXV203 [6]) onto th [file pbio.1001432.s003.tif]

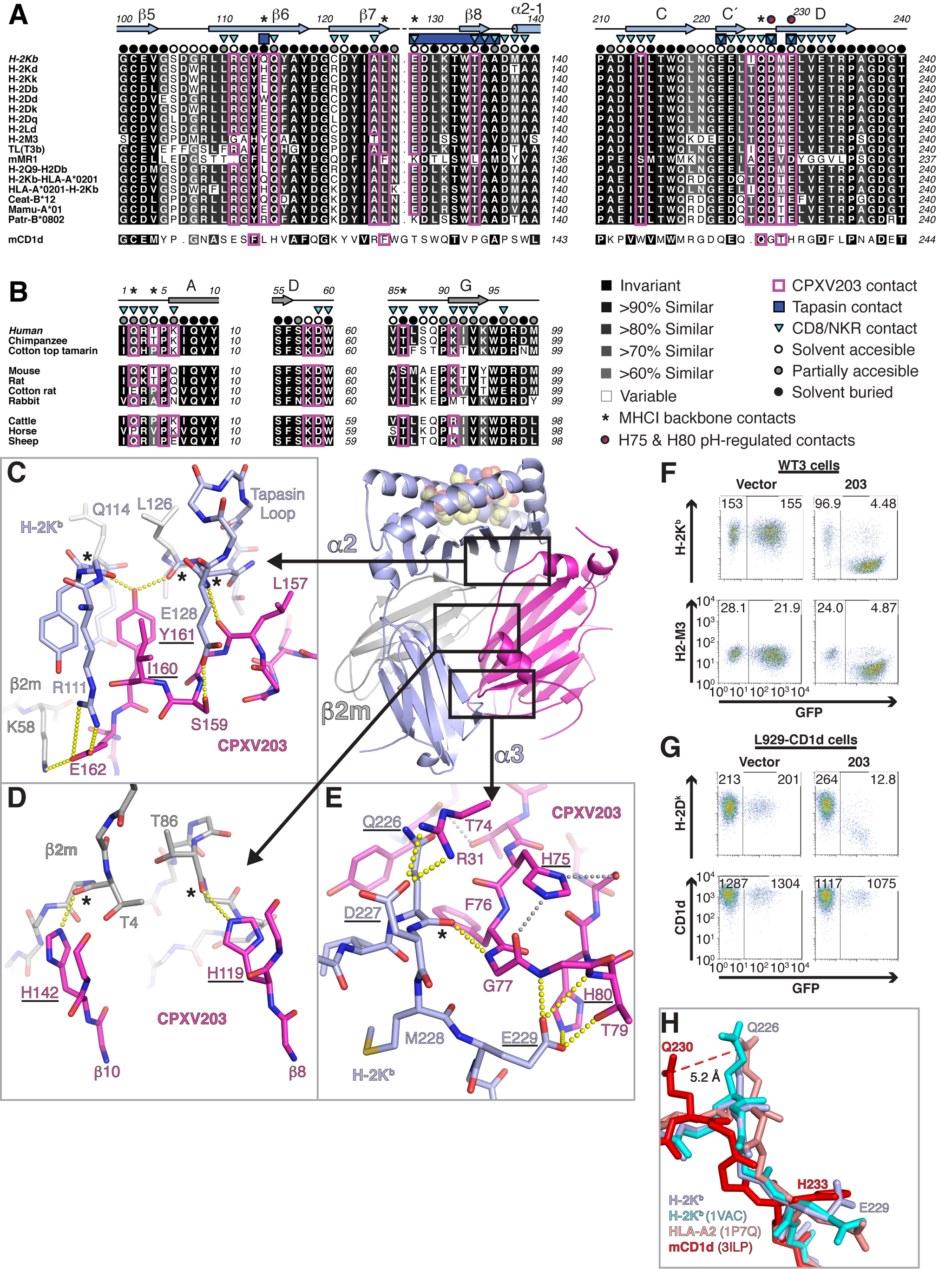

Supplement: Figure S4 — CPXV203/MHCI interface analysis. (A,B) Sequence alignment of MHCI regions contacted by CPXV203. MHCI 2o structure, Tpn/CD8/NKR contacts, and side chain solvent accessibility are shown above the alignments (see Figure S3 for additional alignment details). CPXV203/MHCI contacts are boxed in magenta, while MHCI backbone contacts are indicated with an asterisk above the alignment. (A) MHCI HC were included in this alignment if they are downregulated or bound by CPXV203. Murine CD1d was identified as a null allele in this study (see G), and so it is shown below the other alleles to highlight the lack of sequence conservation in CPXV203/MHCI contact regions. D227 and E229 are indicated with blue/red circles, as CPXV203 H75 and H80 are believed to contact these Tpn contact residues in a pH-regulated manner. (B) β2m sequences of potential poxvirus hosts are shown (primate, rodent, livestock). Tpn contacts are based on published mutagenesis work [7]–[10], while HBPLUS was used to identify CD8 and NKR contacts from all available complex structures (CD8/MHCI: 1AKJ, 1BQH, 1NEZ, 3DMM; Ly49/MHCI: 1QO3, 3C8K; LIR/MHCI: 1P7Q, 2DYP) [11]–[19]. (C–E) The CPXV203/MHCI complex is shown using stick representation with each interface colored by chain (as in Figure 3A) and element (C, blue/grey/magenta; O, red; N, dark blue). Side-chains are shown for all contact residues, and MHCI backbone contacts are indicated (*). Residues that appear to be involved in putative hydrogen-bond networks (interface contacts: yellow dots; potential β5–β6 loop conformation stabilizers: grey dots) are labeled. Interface views were rotated (relative to Figure 3A) for optimal viewing: C 10° (y); D 90° (y); E ∼90° (x), ∼150° (z). (C) CPXV203 contacts the underside of the MHCI peptide-binding platform (α1/α2) through burial of large hydrophobic residues (I160 and Y161) immediately following helix-5 (h5). (D) Parallel CPXV203 histidines from β-sheets I and II coordinate β2m backbone carbonyl oxygens. The β2m inte [file pbio.1001432.s004.tif]
