# Supplementary material for: Structural Mechanism of ER Retrieval of MHC Class I by Cowpox
Source: PLoS Biol. 2012 Nov 27;10(11):e1001432. doi: 10.1371/journal.pbio.1001432 (PMC3507924; doi:10.1371/journal.pbio.1001432)
Supplement: Table S1 — SPR analysis of CPXV203/MHCI binding. (DOCX) [file pbio.1001432.s005.docx]

| **pH** | **Heavy Chain** | **β2m** | **K_D,Eq_ (R_Eq_) (nM)** | **K_D,Kin_ (k_d_/k_a_) (nM)** | **k_a_ (M^-1^s^-1^) x 10^5^** | **k_d_ (s^-1^) x 10^-2^** | **t_1/2_ (s)** | **χ^2^** |
| --- | --- | --- | --- | --- | --- | --- | --- | --- |
| 7.4 | H-2K^b^ | human | 2300 (±200)^c,d^ | 480 (±70)^c^ | 2.5 (±0.4) | 12 (±4) | 6 (±2) | 4.9/0.18 |
| 7.4 | H-2D^b^ | human | 730 (±20) | 200 (±10) | 3.7 (±0.2) | 7.7 (±0.1) | 9.0 (±0.2) | 2.0/0.80 |
| 7.4 | H-2D^q^ | human | ≥1400 (±200)^e^ | 97 (±2) | 8.2 (±0.1) | 7.93 (±0.06) | 8.74 (±0.07) | 0.8/0.13 |
| 7.4 | H-2K^d^ | human | ≥12850 (±20)^e^ | 6600 (±400) | 1.18 (±0.06) | 78.4 (±0.8) | 0.884 (±0.009) | 0.03/0.10 |
| 7.4 | H-2L^d^ | human | 390 (±20) | 82 (±2) | 8.2 (±0.1) | 6.67 (±0.09) | 10.4 (±0.1) | 2.3/0.50 |
| 7.4 | TL (T3b) | human | 2200 (±200) | 630 (±40) | 0.88 (±0.04) | 5.5 (±0.1) | 12.6 (±0.3) | 0.7/0.13 |
| 7.4 | Ceat-B*-12^a^ | human | ≥30000 (±6000)^e^ | 10500 (±100) | 0.945 (±0.008) | 98.9 (±0.4) | 0.701 (±0.003) | 0.13/0.17 |
| 6.0 | H-2K^b^ | murine | -^f^ | 64 (±1) | 17.4 (±0.2) | 11.02 (±0.08) | 6.29 (±0.04) | 0.24 |
| 6.0 | H-2K^b^ | human | - | 10 (±2)^c^ | 10 (±2) | 1.0 (±0.1) | 73 (±9) | 0.13 |
| 6.0 | H-2D^k^ | murine | - | 17.8 (±0.4) | 35.1 (±0.4) | 6.26 (±0.06) | 11.1 (±0.1) | 0.20 |
| 6.0 | H-2D^k^ | human | - | 7.20 (±0.09) | 36.1 (±0.3) | 2.60 (±0.01) | 26.7 (±0.1) | 0.18 |
| 6.0 | H-2K^k^ | human | - | 8.2 (±0.5) | 67 (±2) | 5.5 (±0.2) | 12.7 (±0.4) | 0.27 |
| 6.0 | H-2D^d^ | human | - | 4.56 (±0.07) | 61.6 (±0.5) | 2.81 (±0.02) | 24.7 (±0.2) | 0.17 |
| 6.0 | H-2K^d^ | human | - | 10.0 (±0.2) | 20.7 (±0.2) | 2.06 (±0.02) | 33.7 (±0.3) | 0.21 |
| 6.0 | Mamu-A*01^a^ | human | - | 22.2 (±0.9) | 38.3 (±0.7) | 8.5 (±0.2) | 8.1 (±0.2) | 0.16 |
| 6.0 | Patr-B*0802^a^ | human | - | 6.28 (±0.07) | 54.7 (±0.3) | 3.43 (±0.02) | 20.2 (±0.1) | 0.26 |
| 6.0 | H2-Q9-H-2D^b^ | human | - | 18.0 (±0.8) | 53 (±1) | 9.6 (±0.2) | 7.2 (±0.2) | 0.17 |
| 6.0 | H-2K^b^-HLA-A^b^ | human | - | 6.03 (±0.07) | 24.2 (±0.1) | 1.458 (±0.008) | 47.5 (±0.2) | 0.23 |
| 6.0 | HLA-A^b^-H-2K^b^ | human | - | 15.2 (±0.7) | 17.0 (±0.5) | 2.57 (±0.05) | 27.0 (±0.5) | 0.17 |

**Table S1. SPR analysis of CPXV203/MHCI binding.**

SPR assays were run at pH_ER_ 7.4 and pH_Golgi_ 6.0 in triplicate (≥8 curves/K_D,Eq_, ≥5 curves/K_D,Kin_) on a Biacore T100 and fit to a 1:1 Langmuir binding model. Mammalian CPXV203 was used unless otherwise noted.

^a^Primate alleles.

^b^Human allele HLA-A*0201.

^c^Similar constants obtained for mammalian & bacterially produced CPXV203.

^d^Average from experiments on multiple days with multiple protein batches.

^e^10X K_D,Eq_ was not reached for these alleles.

^f^Sigmoidal binding at low pH (see SUPPLEMENTAL METHODS) negated the use of a simple 1:1 Langmuir equilibrium binding model.
