# Supplementary material for: Structural Mechanism of ER Retrieval of MHC Class I by Cowpox
Source: PLoS Biol. 2012 Nov 27;10(11):e1001432. doi: 10.1371/journal.pbio.1001432 (PMC3507924; doi:10.1371/journal.pbio.1001432)
Supplement: Table S2 — Summary of data collection, phasing, and refinement. (DOCX) [file pbio.1001432.s006.docx]

| Data Collection for CPXV203/OVA:H-2K^b^:hβ2m^a^ |  |
| --- | --- |
| Space Group | P 1 |
| Cell dimensions |  |
| a, b, c (Å) | 88.31, 88.25, 106.42 |
| α, β, γ (^o^) | 76.18, 69.29, 66.69 |
| Data Set | SeMet |
| Wavelength (Å) | 0.97909 |
| X-ray Source | ALS 4.2.2^b^ |
| Resolution (Å) overall (outer shell) | 50-3.0 (3.19-3.00) |
| Observations/Unique | 198533/47950 |
| Multiplicity | 4.1 (4.2) |
| Completeness (%) | 87.9 (84.9) |
| R-sym (%) | 16.4 (62.2) |
| I/σ | 7.3 (2.6) |
| Refinement Statistics^c^ |  |
| Complexes in ASU | 4 |
| Reflections Rwork/Rfree | 47881/2394 |
| #Residues/Protein Atoms/Solvent | 2284/18596/72 |
| Rwork overall (outer shell) (%) | 22.9 (31.6) |
| Rfree overall (outer shell) (%) | 25.3 (35.5) |
| RMSD bond lengths (Å)/angles (^o^) | 0.002/0.538 |
| RMSD Dihedral (^o^) | 9.662 |
| Ramachandran plot^d^ |  |
| Favored/Allowed/Outliers (%) | 97.2/2.8/0.0 |
| Estimated Coordinate Error (Å) | 0.46 |
| Structural Analysis |  |
| Clashscore^d^ | 8.56 |
| Bound vs. free MHCI Cα RMSD (Å)^e^ |  |
| (HC, α1/α2, α3, β2m*, OVA) | 1.61, 0.42, 0.71, 0.45*, 0.28 |

**Table S2. Summary of Data Collection, Phasing, and Refinement.**

^a^Values as defined in HKL2000 [53].

^b^Advanced Light Source, Beamline 4.2.2

^c^Values as defined in PHENIX [56].

^d^MolProbity scores output within PHENIX.

^e^Reference PDBs: 1VAC (H-2K^b^:mβ2m), *2F74 (H-2D^b^:hβ2m).
