# Supplementary material for: Structural Mechanism of ER Retrieval of MHC Class I by Cowpox
Source: PLoS Biol. 2012 Nov 27;10(11):e1001432. doi: 10.1371/journal.pbio.1001432 (PMC3507924; doi:10.1371/journal.pbio.1001432)
Supplement: Table S3 — Structural comparison of CPXV203 with vCCI-like CKBPs. (DOCX) [file pbio.1001432.s007.docx]

| **Protein (Virus)** | **RMSD^a^ (Å^2^)** | **Aligned Residues** | **Identity^a^ (%)** | **Conserved Disulfides** | **Nascent length** | **Mature length** | **Localization Signal** |
| --- | --- | --- | --- | --- | --- | --- | --- |
| CPXV203 (CPXV) | - | - | - | 5/5 | 225 | 209 | KTEL |
| CrmD-CTD (CPXV)^b^ | 2.8 | 136 | 12 | 3/5 | 320^b^ | 300^b^ | - |
| A41 (VACV) | 3.9 | 134 | 15 | 4/5 | 219 | 199 | - |
| vCCI (ECTV) | 3.0 | 143 | 14 | 4/5 | 247 | 231 | - |
| vCCI (RPV) | 3.1 | 139 | 15 | 4/5 | 258 | 242 | - |
| vCCI (CPXV) | 3.1 | 139 | 14 | 4/5 | 246 | 224 | - |

**Table S3. Structural comparison of CPXV203 with vCCI-like CKBPs.**

**General comparison**

**Electrostatics and 2^o^ structure comparison**

| **Protein (Virus)** | **CKB Patch** | **CKB loop (2-3)** |  | **7-9 Junction** |  | **vCCI β13-β14** |  |
| --- | --- | --- | --- | --- | --- | --- | --- |
|  | **β-sheet II^c^** | **Charge^c^** | **Length^d^** | **Length^d^** | **β-strand^d,e^** | **(α/β)^^** | **Length^d^** |
| CPXV203 (CPXV) | Neutral | Neutral | 6: R38-G43 | 19: D110-Y128 | 9: F118-S126 | α | 29: E152-L180 |
| CrmD-CTD (CPXV)^g^ | Neutral | Neutral | 3: I184-S186 | 20: N247-H266 | 4: S252-L255 | β | 27: N288-P314 |
| A41 (VACV) | NEGATIVE | Neutral | 2: K39-Y40 | 32: E113-M144 | - | β | 32: E165-F196 |
| vCCI (ECTV) | NEGATIVE | NEGATIVE | 15: S52-P66 | 29: S140-S168 | - | β | 37: M191-L227 |
| vCCI (RPV) | NEGATIVE | NEGATIVE | 25: E53-P77 | 29: S151-I179 | - | β | 37: M202-L238 |
| vCCI (CPXV) | NEGATIVE | NEGATIVE | 14: E46-P59 | 29: S133-I161 | - | β | 37: M184-L220 |

Structures used in this analysis: 3ONA, 2VGA, 2GRK, 2FFK, 1CQ3. Chain A from each structure was used in this analysis.

^a^The Dali server identified poxvirus CKB proteins as structurally similar to CPXV203. RMSD and identity are derived from Dali structural alignment.

^b^C-terminal domain (CTD) of ECTV CrmD (fusion of TNFR to SECRET domain).

^c^The electrostatic surface for each molecule was evaluated using APBS within PyMOL.

^d^Length:boundaries; 2^o^ structure defined by STRIDE.

^e^β-strand not present in vCCI-like proteins that extends β-sheet II.

^f^Primary 2^o^ structure within this region.

^g^Unlike the other proteins, the CrmD-CTD authors [1] used nascent protein residue numbering.
