# Supplementary material for: Structural Mechanism of ER Retrieval of MHC Class I by Cowpox
Source: PLoS Biol. 2012 Nov 27;10(11):e1001432. doi: 10.1371/journal.pbio.1001432 (PMC3507924; doi:10.1371/journal.pbio.1001432)
Supplement: Table S4 — CPXV203/MHCI interactions. (DOCX) [file pbio.1001432.s008.docx]

**Table S4. CPXV203/MHCI interactions.**

**Hydrogen bonds^a^**

| **Structural Element^b^** | **CPXV203** | **H-2K^b^ α2 domain** | **Length (Å)** |
| --- | --- | --- | --- |
| h4 | L157 O | E128 N | 3.1 |
| loop^h4-h5^ | S159 Oγ | E128 Oε2 | 2.3 |
|  | S159 N | E128 Oε2 | 3.4 |
|  | Y161 OH | Q114 N | 3.3 |
|  | Y161 OH | L126 O | 2.6 |
|  | E162 Oε1 | R111 Nη1 | 4.9 (LRE)^c^ |
|  | E162 Oε2 | R111 Nη1 | 3.5 (LRE)^c^ |
|  |  | **H-2K^b^ α3 domain** |  |
| loop^b1-b2^ | R31 Nη1 | D227 Oδ1 | 2.9 |
|  | R31 Nη2 | D227 Oδ1 | 2.9 |
| loop^b5-b6^ | G77 N | Q226 O | 2.7 |
|  | V78 N | E229 Oε1 | 2.6 |
|  | T79 Oγ1 | E229 Oε2 | 2.4 |
|  | T79 N | E229 Oε1 | 3.3 |
| b6 | H80 Nε2^d^ | E229 Oε2 | 3.5 |
|  |  | **β2m** |  |
| b8 | H119 Nε2 | T86 O | 3.0 |
|  | Y123 O | Q2 N | 3.2 |
|  | E125 N | M0 O^e^ | 2.8 |
|  | E125 O | M0 N^e^ | 2.4 |
| b10 | N141 Oδ1 | K91 Nζ | 3.1 |
|  | H142 Nε2 | T4 O | 2.7 |
| loop^h4-h5^ | E162 Oε1 | K58 Nζ | 2.9 |
|  | T165 Oγ1 | D59 Oδ2 | 2.9 |

**Contacts <4.0 Å^a,f^**

| **Structural Element^b^** | **CPXV203** | **H-2K^b^ α2 domain** |
| --- | --- | --- |
| h4 | E154 (2) | T134 |
| loop^h4-h5^ | I160 (2) | E128 |
|  | Y161 (5) | Y113, Q115 (3), A125 |
|  |  | **H-2K^b^ α3 domain** |
| loop^b1-b2^ | R31 (1) | Q226 |
| b5 | F76 (3) | Q226 |
| loop^b5-b6^ | G77 (2) | M228, E229 |
| loop^h4-h5^ | K171 (1) | T214 |
| h5 | T175 (1) | I225 |
|  | Y179 (6) | I225 (2), Q226 (4) |
|  |  | **β2m** |
| b8 | L121 (2) | T4, T86 |
| b10 | H142 (2) | K6 |

^a^Contacts calculated with HBPLUS.

^b^Structural element refers to CPXV203.

^c^(LRE) = Long range electrostatics (3.5-5.0 Å) calculated with NCONT.

^d^Identified by model inspection.

^e^Artifact contacts due to use of refolded hβ2m.

^f^Number of non-bonded contacts listed in parentheses.
