# Supplementary material for: Structural Mechanism of ER Retrieval of MHC Class I by Cowpox
Source: PLoS Biol. 2012 Nov 27;10(11):e1001432. doi: 10.1371/journal.pbio.1001432 (PMC3507924; doi:10.1371/journal.pbio.1001432)
Supplement: Table S5 — Buried surface area comparison of CPXV203/MHCI to similar interfaces. (DOCX) [file pbio.1001432.s009.docx]

**Table S5. Buried surface area comparison of CPXV203/MHCI to similar interfaces.**

|  | **CPXV203/H-2K^b^** | **US2/HLA-A2** | **CD8αα/H-2K^b^** | **CD8αβ/H-2D^d^** | **Ly49A/H-2D^d^** | **Ly49C/H-2K^b^** | **LIR-1/HLA-A2** |
| --- | --- | --- | --- | --- | --- | --- | --- |
| Receptor/MHCI | 3342 (558) | 1272 (223) | 2936 (270) | 1501 (149) | 3343 (343) | 2368 (222) | 1712 (315) |
| Receptor | 1668 (263) | 628 (85) | 1477 (147) | 705 (62) | 1729 (149) | 1217 (92) | 864 (144) |
| MHCI | 1675 (295) | 645 (138) | 1460 (123) | 797 (87) | 1614 (194) | 1152 (130) | 848 (171) |
| β2m | 565 (125) | 0 (0) | 233 (10) | 7 (0) | 456 (59) | 281 (20) | 566 (129) |
| HC | 1110 (170) | 645 (138) | 1226 (114) | 790 (87) | 1159 (135) | 871 (110) | 282 (43) |
| Platform | 566 (77) | 270 (72) | 133 (0) | 25 (0) | 934 (130) | 555 (63) | 0 (0) |
| α1 | 5 (0) | 24 (19) | 57 (0) | 0 (0) | 298 (61) | 20 (0) | 0 (0) |
| α2 | 560 (77) | 246 (53) | 76 (0) | 25 (0) | 636 (69) | 535 (63) | 0 (0) |
| α3 | 544 (93) | 375 (66) | 1093 (114) | 765 (87) | 224 (5) | 316 (47) | 282 (42) |
| Tpn/CD8 α3 ridge | 441 (86) | 0 (0) | 796 (101) | 543 (76) | 128 (5) | 152 (27) | 5 (0) |
| Affinity (pH 7.4) | 480 nM | Low nM^a^ | ≥35000 nM^a^ | 8200 nM^a^ | ≥6000 nM^a^ | ≥80000 nM^a^ | >3000 nM^a^ |
| (pH 6.0) | 10 nM | - | - | - | - | - | - |

Buried surface areas (BSA) were calculated with NACCESS (Hubbard and Thornton, 1993) using a 1.4 Å probe. BSA results are listed as TOTAL (MAIN-CHAIN) Å^2^. MHCI domains: α1 (1-90), α2 (91-180), α3 (181-274). Tpn/CD8 α3 ridge (220-233) includes CD loop and part of strand D.

β2m (M0) is present in all complexes. The measured affinity or an estimate (grey text) based on available literature is listed for each complex.

PDB coordinates used in this table: 1IM3, 1BQH, 3DMM, 1QO3, 3C8K, 1P7Q.

^a^Please see SUPPLEMENTAL METHODS for references for these binding constants.
