# Supplementary material for: Structural Mechanism of ER Retrieval of MHC Class I by Cowpox
Source: PLoS Biol. 2012 Nov 27;10(11):e1001432. doi: 10.1371/journal.pbio.1001432 (PMC3507924; doi:10.1371/journal.pbio.1001432)
Supplement: Table S6 — BLI analysis of mutant CPXV203/MHCI binding. (DOCX) [file pbio.1001432.s010.docx]

| **pH** | **CPXV203^a^** | **H-2K^b^** | **β2m^b^** | **K_D,Eq_ (nM)** | **R^2^** | **[CPXV203]_max_ (nM)** | **Res Selection^c^** | **mt/wt^d^** |
| --- | --- | --- | --- | --- | --- | --- | --- | --- |
| 7.4 | wt | wt | h(wt) | 1300 (±700) | 0.99 (±0.02) | 23000 | --- | --- |
| 7.4 | H75A | wt | h(wt) | 2270 (±60) | 0.993 (±0.003) | 26000 | Contact | 1.7 |
| 7.4 | F76A^e^ | wt | h(wt) | >27000 (±3000) | 0.993 (±0.003) | 250000 | Contact | >20 (🡻) |
| 7.4 | G77A^e^ | wt | h(wt) | >110000 (±20000) | 0.98 (±0.02) | 150000 | Contact | >86 (🡻) |
| 7.4 | H80A | wt | h(wt) | 5700 (±300) | 0.974 (±0.003) | 200000 | Contact | 4.3 (🡻) |
| 7.4 | H75A,H80A | wt | h(wt) | 10000 (±900) | 0.991 (±0.003) | 250000 | Contact | 7.5 (🡻) |
| 7.4 | wt | G56R | h(wt) | 2130 (±60) | 0.997 (±0.001) | 37000 | E3-19K mutation | 1.6 |
| 7.4 | wt | N86A | h(wt) | 1870 (±60) | 0.990 (±0.006) | 37000 | CRT site | 1.1 |
| 7.4 | wt | T134K | h(wt) | 7000 (±2000) | 0.97 (±0.03) | 300000 | Tpn site | 5.1 (🡻) |
| 7.4 | wt | M138K | h(wt) | 2000 (±100) | 0.995 (±0.006) | 300000 | MAb Y-3 site | 1.5 |
| 7.4 | wt | L141R | h(wt) | 1500(±200) | 0.97 (±0.03) | 300000 | MAb Y-3 site | 1.2 |
| 7.4 | wt | A150P | h(wt) | 3000 (±1000) | 0.985 (±0.006) | 300000 | MAb Y-3 site | 2.3 |
| 7.4 | wt | R181E | h(wt) | 2300 (±300) | 0.989 (±0.009) | 300000 | US2 mutation | 1.8 |
| 7.4 | wt | D227K | h(wt) | 7000 (±2000) | 0.992 (±0.003) | 300000 | Tpn site | 4.9 (🡻) |
| 7.4 | wt | Y84A,C121S | h(wt) | 317 (±6) | 0.99 (±0.01) | 37000 | --- | (🡹) 0.2 |
| 7.4 | wt | Y84C,C121S^f^ | h(wt) | 260 (±20) | 0.997 (±0.002) | 37000 | --- | (🡹) 0.2 |
| 7.4 | wt | wt | h(I1R) | 20000 (±2000) | 0.988 (±0.002) | 300000 | Contact | 15 (🡻) |
| 7.4 | wt | wt | h(R3E) | 10000 (±2000) | 0.987 (±0.005) | 300000 | Near interface | 7.6 (🡻) |
| 7.4 | wt | wt | h(EEY)^g^ | 27000 (±2000) | 0.981 (±0.007) | 300000 | Contact | 20 (🡻) |

**Table S6. BLI analysis of mutant CPXV203/MHCI binding.**

BLI assays were run at pH_ER_ 7.4 in triplicate (≥8 curves/K_D,Eq_) using an Octet RED and fit using non-linear least squares analysis.

^a^Refolded CPXV203(0-205) was used in these assays.

^b^h(wt/---) = human wild-type/mutant β2m.

^c^Selection criteria for each mutation.

^d^(K_D,Eq mt_)/(K_D,Eq wt_); significant affinity changes (>3-fold) are indicated with arrows (🡹= increase, 🡻= decrease).

^e^10X K_D,Eq_ was not reached for these MHCI.

^f^Disulfide-trapped MHCI (H-2K^b^:OVAgc).

^g^β2m amino acids 57-59 (SKD) were mutated to EEY.
