# Supplementary material for: Structural Mechanism of ER Retrieval of MHC Class I by Cowpox
Source: PLoS Biol. 2012 Nov 27;10(11):e1001432. doi: 10.1371/journal.pbio.1001432 (PMC3507924; doi:10.1371/journal.pbio.1001432)
Supplement: Table S7 — SPR analysis of mutant CPXV203/MHCI binding. (DOCX) [file pbio.1001432.s011.docx]

| **pH** | **CPXV203** | **H-2K^b^** | **β2m** | **K_D,Eq_ (R_Eq_) (nM)** | **K_D,Kin_ (k_d_/k_a_) (nM)** | **k_a_ (M^-1^s^-1^) x 10^5^** | **k_d_ (s^-1^) x 10^-2^** | **t_1/2_ (s)** | **χ^2^** |
| --- | --- | --- | --- | --- | --- | --- | --- | --- | --- |
| 7.4 | wt | wt | h(wt) | 2300 (±200)^a,b^ | 480 (±70)^a^ | 2.5 (±0.4) | 12 (±4) | 6 (±2) | 4.9/0.18 |
| 7.4 | H75A | wt | h(wt) | 3300 (±800) | 1400 (±200) | 0.53 (±0.05) | 7.40 (±0.05) | 9.37 (±0.06) | 0.09/0.13 |
| 7.4 | H80A | wt | h(wt) | 2800 (±200) | 1300 (±400) | 0.6 (±0.1) | 7.6 (±0.5) | 9.2 (±0.7) | 0.07/0.23 |
| 7.4 | wt | N127K | h(wt) | 5500 (±500) | 550 (±10) | 3.15 (±0.05) | 17.5 (±0.1) | 3.97 (±0.03) | 17/0.15 |
| 7.4 | wt | K131D | h(wt) | 2500 (±300) | 291 (±5) | 3.15 (±0.04) | 9.14 (±0.06) | 7.58 (±0.05) | 13/0.41 |
| 6.0 | wt | wt | h(wt) | -^c^ | 10 (±2) ^a^ | 10 (±2) | 1.0 (±0.1) | 73 (±9) | 0.13 |
| 6.0 | H75A | wt | h(wt) | - | 210 (±70) | 4 (±1) | 8 (±2) | 9 (±3) | 0.12 |
| 6.0 | H80A | wt | h(wt) | - | 220 (±70) | 3.3 (±0.6) | 7 (±3) | 11 (±4) | 0.16 |
| 6.0 | wt | N127K | h(wt) | - | 7.1 (±0.2) | 41.0 (±0.6) | 2.90 (±0.04) | 23.9 (±0.3) | 0.10 |
| 6.0 | wt | K131D | h(wt) | - | 4.03 (±0.06) | 36.0 (±0.3) | 1.45 (±0.009) | 47.8 (±0.3) | 0.14 |

**Table S7. SPR analysis of mutant CPXV203/MHCI binding.**

SPR assays were run at pH_ER_ 7.4 and 6.0_Golgi_ in triplicate (≥8 curves/K_D,Eq_, ≥6 curves/K_D,Kin_) on a Biacore T100 and fit to a 1:1 Langmuir

binding model. wt = wild-type, h(wt) = human wild-type β2m.

^a^Similar constants obtained for mammalian & bacterially produced CPXV203.

^b^Average from experiments on multiple days with multiple protein batches.

^c^Sigmoidal binding at low pH (see SUPPLEMENTAL METHODS) negated the use of a simple 1:1 Langmuir equilibrium binding model.
