# Supplementary material for: Structural Mechanism of ER Retrieval of MHC Class I by Cowpox
Source: PLoS Biol. 2012 Nov 27;10(11):e1001432. doi: 10.1371/journal.pbio.1001432 (PMC3507924; doi:10.1371/journal.pbio.1001432)
Supplement: Text S1 — Supplemental methods. (DOCX) [file pbio.1001432.s012.docx]

**SUPPLEMENTAL METHODS**

**MAb Footprint Assignment**

The TCR-like MAb 25-D1.16 (IgG1) specifically binds H-2K^b^ loaded with OVA_257-264_ (SIINFEKL) [23–25]. MAb B8-24-3 (IgG1) is specific for folded H-2K^b^, it binds around the C-terminus of the α1 helix, and it is sensitive to R75H, L82(P/F), & K89A mutations [26–29]. MAb Y-3 [30] (IgG2a) is sensitive to H-2K^b^ α2-1 helix mutations (A150P, L141R, M138K) [31], insensitive to epitope destruction via formaldehyde [32], and competes with MAbs 25-D1.16, B8-24-3, & AF6-88.5.3 (unpublished SPR results). MAb AF6-88.5.3 [33] (IgG2a) binds to folded H-2K^b^/mβ2m below the α2-1 helix, as indicated by hβ2m substitution [34], sensitivity to domain swaps (K^b^🡺L^d^) [35], competition with Y-3 (unpublished SPR results), and sensitivity to M228T mutation (unpublished SPR results). MAbs 28-8-6 (IgG2a) & 34-1-2S (IgG2a) recognize folded H-2K^b^/D^b^ & H-2K^d^/D^d^/K^b^ (α3 domain), respectively. MAb 2M2 (IgG1) recognizes a linear determinant that is available on native hβ2m, bβ2m, but not mβ2m (unpublished SPR results). MAb 5F1-2-14 recognizes folded H-2K^b^ and is sensitive to α2-2 helix mutations G162D, E166K, & N174K [31].

**Peptides**

Peptides were synthesized either for *in vitro* MHCI refolding or for exogenous peptide loading of MHCI *in vivo*. Each peptide listed indicates the targeted HC, peptide name, peptide sequence, and literature reference used. Peptides without an indicated reference were suggested by A. Stout (NIH Tetramer Core Facility). The peptides used in this study are as follows: H-2K^b^ (OVA_257-264_, SIINFEKL) [21], H-2K^d^ (TYQRTRALV, Flu NP_147–155_) [36], H-2K^k^ (SV40_560–568_, SEFLLEKRI) [37], H-2D^d^ (Env-modified PA9, IGPGRAFYA) [38], H-2D^k^ (Middle T-antigen_389-397_, RRLGRTLLL), H-2D^q^ (Her2_395-404_, PDSLRDLSVF), H-2L^d^ (p29, YPNVNIHNF) [39], Ceat-B*12 (nef_20-28_, LLRARGETY), H2-M3 (Fr38, fFMIVIL) [20], Mamu-A*01 (Tat_28–35_, TTPESANL) [40], Patr-B*0802 (Polyprotein_1527-1536_, AGCAWYELTP), H-2Q9-H-2D^b^ (Minor capsid protein VP3_24-32_, HALNVVHDW), H-2K^b^-HLA-A*0201 (OVA_257-264_, SIINFEKL) [21], HLA-A*0201-H-2K^b^ (Insulin_2-10_, ALWMRLLPL).

**Protein cloning, expression, and purification**

CPXV203ΔKTEL (aa 1-205, MVI-LHV) was amplified from Cowpox virus (Brighton Red) genomic DNA (kindly provided by R. M. L. Buller), ligated into the mammalian expression vector pHLsec (AgeI-KpnI), expressed via large-scale transient transfection (HEK293F) [41], and then purified using NiNTA🡺SEC. Mammalian CPXV203ΔKTEL was also produced using its authentic signal peptide (aa -16-205, MRS-LHV) to verify the signal peptide cleavage site (IYS-MVI) predicted by SignalP3.0 [42]. Bacterial CPXV203ΔKTEL (maMVI-LHV) & CPXV203-BirA (maMVI-LHV-[Thr-BirA-6His] [43]) were produced from inclusion bodies (IBs) using established methods (pET28a [44], autoinduction [45], arginine refolding [46]) followed by IEX🡺SEC🡺HIC. A truncated, soluble CPXV203 domain (aa (0)5-190, mRCE-FDR) identified using limited proteolysis followed by mass spec (LPMS) [47] was also refolded from IBs. SDS PAGE, mass spec (24955, ND, 23838 (SeMet-labeled), 27361, ND), and N-terminal sequencing (etgMV…, MVIRR…, aMVIR…, ND, ND) confirmed the expected MW & construct boundaries for mammalian (no glycans indicated) & bacterial CPXV203 constructs. Recombinant MHCI were produced by refolding bacterial IBs as previously described [46]. H-2K^b^ (aa 0-280, mGPH-PST), H-2K^d^ (aa 0-283, mGPH-VSN), and disulfide-trapped MHCI (H-2K^b^:OVAgc) [48] constructs were produced in house, while H-2D^d^, H-2D^k^, H-2D^q^, H-2K^k^, H-2L^d^, Mamu-A*01, Patr-B*0802, H-2Q9-H-2D^b^, H-2K^b^-HLA-A*0201, HLA-A*0201-H-2K^b^ were produced by the NIH Tetramer Core Facility. Chimeric MHCI from the NIH include α1-α2 of the first allele and α3 of the second allele. H-2D^b^ biotinylated monomer (LCMV Gp33, KAVYNFATC) was purchased from Beckman Coulter. Chimeric mCD1d-Fc was obtained from R&D Systems. All MHCI include human β2m (hβ2m, aa 0-99, mIQR-RDM) unless otherwise noted. MHCI captured via neutravidin in biosensor assays include a C-terminal biotin ligase site and were biotinylated in vitro using established methods (Avidity). Mutations were introduced using QuikChange (Stratagene) or Phusion (NEB) site-directed mutagenesis kits. CPXV203/MHCI proteins (wt & mutants) were purified to >95% homogeneity and produced single, monodispersed SEC peaks (4 ^o^C; HEPES sizing buffer: 150 mM NaCl, 10 mM HEPES pH 7.5, 0.01% Azide) corresponding to the MW of a monomer/heterotrimer, respectively. Recombinant proteins demonstrated expected 2^o^ structure by circular dichroism (CD) (data not shown). Selenomethionine (SeMet)-labeled protein were produced in E. coli using autoinduction [45] and >90% incorporation was verified by mass spec. Signal-peptide/cloning artifacts are indicated as lower-case aa. UniProt (UNP) accession codes for proteins used in this work include CPXV203 (Q8QMP2), H-2K^b^ (P01901), H-2K^d^ (Q6RJ37), H-2D^d^ (P01900), H-2D^k^ (P14426), H-2D^q^ (Q3KQJ3), H-2K^k^ (P04223), H-2L^d^ (P01897), Mamu-A*01 (Q30596), Patr-B*0802 (Q9MXI2), H-2Q9 (P14431), HLA-A*0201 (Q8WLS4), human β2m (P61769), and murine β2m (Q91XJ8).

**Crystallization & data collection**

Exhaustive attempts to crystallize CPXV203 and the T4 protein from Monkeypox (MPX-Zaire-1979) MPX184 (64% identity) alone were unsuccessful (mammalian/bacterial, reductive methylation [49]/pentylation [50], in situ proteolysis [51], LPMS [47] truncations native/methylated/pentylated), while complex crystals (CPXV203/H-2K^b^:mβ2m) grew in a variety of conditions possibly due to favorable MHCI/MHCI crystal contacts. Unfortunately, all complex crystals were plagued by extremely poor diffraction (>20 Å), high mosaicity (>2^o^), and significant anisotropy, thereby requiring optimization of complex constituents (hβ2m instead of mβ2m), purification, crystal growth conditions, streak seeding, and finally dehydration before data quality improved to a point where structure determination could be pursued.

CPXV203(aMVI-LHV, SeMet-labeled)/MHCI(OVA_257-264_:H-2K^b^:hβ2m) complex was prepared for crystallization by incubating an excess (> 4:1) of refolded CPXV203 with MHCI at 4 ^o^C for 30 min in low pH/salt sizing buffer (50 mM NaCl, 30 mM MES pH 5.6, 0.01% Azide), purifying the complex by SEC in the same buffer, concentrating (Vivaspin) to 7-7.5 mg/ml, and finally buffer-exchanging (Pierce Zeba Spin Desalting Column) into crystal wash buffer (20 mM Ammonium acetate pH 6.9, 0.01% azide) supplemented with 0.5 mM OVA_257-264_. Diffraction-quality crystals of CPXV203/MHCI were grown at 20 ^o^C by streak seeding into hanging drops of 0.5 μl complex (7.5 mg/ml) + 0.5 μl reservoir solution (10% PEG 6000, 4% Glucose, 2% Ethylene glycol, 0.1 M tri-K citrate pH 5.55, 0.01% Azide). After crystals grew to 0.025-0.1 mm (3-10 days), they were dehydrated by adding 4.5 μl of dehydration solution #1 (15% PEG 6000, 5% Glucose, 2.5% Ethylene glycol, 0.1 M tri-K citrate pH 5.55, 0.01% Azide) to the drop and then adding dehydration solution #2 (40% PEG 6000, 30% Glucose, 15% Ethylene glycol, 0.1 M tri-K citrate pH 5.55, 0.01% Azide) to the 500 μl reservoir over 2 days (100 μl/6 hr x 2, then 200 μl/6 hr x 3; extensive optimization of published protocols [52]). The final dehydration condition was cryoprotective, so no additional reagents were added prior to liquid N_2_ flash freezing. This protocol improved diffraction from >20 Å to 3-8 Å and greatly reduced mosaicity. X-ray diffraction data was collected at ALS (Advance Light Source) beamline 4.2.2 on the NOIR detector to 3.0 Å resolution. A total of 720 frames (1^o^ oscillation) was collected, though the last 180 frames were excluded from final processing due to radiation damage. Crystals belong to space group *P1* with four CPXV203/MHCI complexes per asymmetric unit (ASU) and a solvent content of 52%. The HKL2000 software package [53] was used to index, integrate, & scale the data to yield a 87.9% complete dataset at 3.0 Å (R-sym = 16.4%, Table S2).

**Structure determination & refinement**

BALBES [54] was used to identify RBM5:H-2K^bm8^:mβ2m (PDB code 2CLZ) [55] as the optimal molecular replacement model for MHCI in the CPXV203/MHCI complex, which was then used (without peptide/H_2_O) to identify four molecular replacement (MR) solutions using Phaser (Phenix suite [56]). Exhaustive attempts to identify a MR solution for CPXV203 proved unsuccessful (poxvirus CKBPs, PHYRE [57] & I-TASSER [58] models), and the anomalous signal strength was not sufficient to initially identify SeMet sites (SOLVE/RESOLVE [56], SHARP [59]) for SAD phasing without the inclusion of MR phasing information (see below). Though initial MR density in the CPXV203 region (identified via MAb blocking studies) was quite poor, extensive cross-crystal averaging [60], using DMMULTI [61], improved it to the point where CPXV203 could be built in Coot [62]. The averaging strategy included 4-fold non-crystallographic symmetry (NCS) averaging of the four CPXV203 regions (initially defined using NCSMASK [61] as a 20 Å sphere at the center of CPXV203 density) and cross-crystal averaging of each MHCI domain (α1/α2, α3, β2m) with the densities from six H-2K^b/bm8^ crystal structures: CD8αα/MHCI (1BQH) [63], TCR/MHCI (1FO0) [64], MHCI (2CLZ) [55], TCR/MHCI (2OL3) [65], Ly49C/MHCI (3C8K) [66], MAb/MHCI (3CVH) [67]. Inclusion of a structure required a visible improvement in CPXV203 density following averaging. Rotation & translation matrices were calculated using SUPERPOSE [68]. Iterative rounds of Coot model building & mask optimization (MAMA [69]) were required to produce the final cross-crystal averaged map for CPXV203 model building, while the initial MHCI models were built using only the MR phases. MR-SAD using AutoSol (Phenix suite [56]) was subsequently used to identify eight of the twenty possible CPXV203 SeMet sites. The addition of this anomalous phase information (FOM 0.604) improved map quality to the point necessary to complete CPXV203/MHCI model building. Simulated annealing composite omit maps (Phenix Autobuild [56]) were used to reduce model-bias throughout model building. Non-crystallographic symmetry (global restraints) was used in the final stages of refinement, as four 1:1 CPXV203/MHCI complexes were present in the asymmetric unit related by two non-symmetrical two-fold axes (RMSD 0.28 Å for all Cα). The CPXV203/MHCI structure was refined (Phenix Refine [56]) to a final R_work_ of 22.9% and R_free_ 25.3% (see Table S2 for complete crystallographic statistics). Overlapmap [70] was used to calculate the real-space correlation coefficients of the CPXV203 model at various stages (MR = 0.53, four-fold NCS = 0.61, cross-crystal averaging = 0.65, final composite omit map = 0.85). PROCHECK [71] was used to assess model quality. The final CPXV203/MHCI model contains four copies each of CPXV203 (R5 – S190), H-2K^b^ (G1–P277), β2m (0M-99M), OVA_257-264_; and 72 total water molecules. The large CPXV203/MHCI interface (3,342 Å^2^) shown in Figure 3A is supported by both MAb blocking and mutagenesis, while a smaller CPXV203/MHCI interface (829 Å^2^) localized to the C-terminus of the peptide-binding groove is believed to be a crystal contact, as MAbs that bind this region do not block CPXV203/MHCI association.

**Structure analysis & figures**

The DALI server [72] was used to identify structural homologues and to create structure-based sequence alignments. Combinatorial extension (CE) [6] in PyMOL (Version 1.2r3pre Schrödinger, LLC.) was used to calculate root mean square deviation (RMSD) values between different proteins/domains. The DYNDOM [73] webserver (DomainSelect) was used to calculate domain motions for MHCI in complex with CPXV203 and free MHCI [11,74]. HBPLUS [75] was used to enumerate H-bonds & atomic contacts (≤4 Å), while NCONT [76] was used to identify long-range electrostatic contacts (3.5-5 Å). NACCESS [77] was used to analyze BSA (1.4 Å probe) and to characterize interfaces as polar/neutral/hydrophobic. APBS [78] was used for electrostatics calculations. ClustalW2 [79] was used for general sequence alignment, while the Scorecons server was used to calculate positional conservation within alignments [80]. Reference structures used in Table S2 were 1VAC [21] and 2F74 [81]. Conservation analysis of CPXV203 contacts across all murine pMHCI used the following 22 MHCI molecules: H-2D^b,d,k,p,q,r^, -2K^b,d,k^, -2L^d^; H2-B1; H2-M3; H2-Q1,2,4-10; H2-T23. Binding constants in Table S5 were obtained from the literature [14,17,82–87]. ALINE [88], TOPDRAW [89], and PyMOL(Version 1.2r3pre Schrödinger, LLC.) were used to create figures.

**Biosensor measurements**

SPR experiments were run on a Biacore T100 (GE Healthcare) in either standard HBS-EP+ (10 mM HEPES (pH 7.4), 150 mM NaCl, 3.4 mM EDTA, 0.005% v/v Triton X-100, 0.01% Azide) or low pH MBS-EP+ (25 mM MES (pH 6.0), 150 mM NaCl, 3.4 mM EDTA, 0.005% v/v Triton X-100, 0.01% Azide). MHCI chips were generated by activating Biacore CM5 chips (0.2 M EDC/50 mM NHS, 5 μl/min, 7 min), coupling 0.1 mg/ml neutravidin (20 mM Na citrate (pH 4.5), 5 μl/min, 7 min), saturating available sites (1 M ethanolamine-HCl (pH 8.0), 5 μl/min, 7 min), washing away non-covalently bound protein (0.1% SDS, 5 μl/min, 1 min), and then capturing site-specifically biotinylated MHCI ligand (equilibrium: <1000 RUs, kinetics: <500 RUs). Flow cell 1 (F1) (neutravidin) was used as the control flow cell, as non-specific binding to neutravidin was observed to be equivalent to neutravidin-captured control proteins (scTCR, WNV DIII, various MAbs). Experiments were performed at 25 ^o^C with a flow rate of 20 μl/min. Proper SPR experimental technique was followed in all assays [90]. Reversing ligand-analyte orientation produced kinetic & equilibrium constants within 2-fold. All binding constants were derived from ≥3 independent experiments. Increasing [HEPES] to 25 mM produced kinetic & equilibrium constants within 2-fold. Equilibrium & kinetic analyses produced similar dissociation constants for CPXV203/MHCI at pH_ER_ 7.4 with differences in the K_D,Eq_ values likely due to a mixed population of CPXV203 histidines containing both protonated and non-protonated forms. As the discrepancy in K_D,Eq_ increased at pH_Golgi_ 6.0, kinetic analysis was used for all assays at pH 6.0 and 7.4. The use of a 1:1 Langmuir binding model in these kinetic analyses was deemed appropriate based on MALS that demonstrated 1:1 stoichiometry from pH 8.5-6.5 (see Figure S1) and DLS that supports 1:1 stoichiometry from pH 7.5-5.4 (data not shown). MAb competition chips were prepared by amine-coupling (CM5) control MAb (Biacore αGST) to F1 and αMHCI MAbs to F2-F4. Competition (direct or steric) was then evaluated by using dual inject over F1-F4: Inj#1, MHCI capture (F2-F4); Inj#2, CPXV203 tetramers or controls (buffer/neutravidin/MAbs). Free MHCI dissociation during Inj#2 indicated competition between capture & detection reagents, while binding during Inj#2 indicated capture & detection do not compete. BLI [91] was used as a separate biophysical approach to evaluate CPXV203/MHCI binding. BLI affinity/kinetic experiments were run on an Octet Red system (ForteBio) using a similar approach to the above SPR assays: HBS-EP+/MBS-EP+ supplemented with 0.05% (v/v) TWEEN & 1% BSA, immobilized MHCI on streptavidin sensors, soluble CPXV203. These assays confirmed the previously observed SPR equilibrium binding constants (within 2-fold). General CPXV203/MHCI pH dependence was evaluated by monitoring the increase in BLI signal (CPXV203 binding) as pH decreased (7.6-6.0) for seven immobilized MHCI proteins **(**H-2D^k^, H-2K^b^, H-2K^k^, H-2L^d^, TL, Mamu-A*01, & Patr-B*0802). This pH range was chosen to mimic the ER🡺TGN pH gradient previously reported in the literature [92–95]. [CPXV203] was held constant, while pH was varied via NaH_2_PO_4_/ Na_2_HPO_4_ ratio (50 mM Na Phosphate, 150 mM NaCl, 3.4 mM EDTA, 0.005% v/v Triton X-100, 0.05% (v/v) TWEEN, 1% BSA 0.05% Azide).

**Light scattering & circular dichroism**

SEC-MALS experiments were run to determine the absolute molecular mass of proteins in solution. These experiments were run on a Dawn HELEOS-II 18-angle light scattering detector (Wyatt) and Optilab rEX refractive index monitor (Wyatt) linked to a Waters HPLC system [96]. DLS experiments were performed using a DynaPro-801TC as previously described [97]. MALS & DLS experiments were run at 20 ^o^C with [protein] = 1 mg/ml. Various buffers (H/M/TBS) were used for MALS & DLS experiments to evaluate the effect of pH on oligomeric state of proteins (alone and in complex). Protein 2^o^ structure was assayed using a Jasco-810 instrument (Jasco Inc., Easton, MD) equipped with Peltier temperature controller using standard methods [98].

**SUPPLEMENTAL REFERENCES**

1. Xue X, Lu Q, Wei H, Wang D, Chen D, et al. (2011) Structural Basis of Chemokine Sequestration by CrmD, a Poxvirus-Encoded Tumor Necrosis Factor Receptor. PLoS Pathog 7: e1002162. doi:10.1371/journal.ppat.1002162.

2. Bahar MW, Kenyon JC, Putz MM, Abrescia NGA, Pease JE, et al. (2008) Structure and function of A41, a vaccinia virus chemokine binding protein. PLoS Pathog 4: e5. doi:10.1371/journal.ppat.0040005.

3. Arnold PL, Fremont DH (2006) Structural determinants of chemokine binding by an Ectromelia virus-encoded decoy receptor. J Virol 80: 7439–7449. doi:10.1128/JVI.00576-06.

4. Zhang L, Derider M, McCornack MA, Jao S-C, Isern N, et al. (2006) Solution structure of the complex between poxvirus-encoded CC chemokine inhibitor vCCI and human MIP-1beta. Proc Natl Acad Sci USA 103: 13985–13990. doi:10.1073/pnas.0602142103.

5. Carfí A, Smith CA, Smolak PJ, McGrew J, Wiley DC (1999) Structure of a soluble secreted chemokine inhibitor vCCI (p35) from cowpox virus. Proc Natl Acad Sci USA 96: 12379–12383.

6. Shindyalov IN, Bourne PE (1998) Protein structure alignment by incremental combinatorial extension (CE) of the optimal path. Protein Eng 11: 739–747.

7. Peace-Brewer AL, Tussey LG, Matsui M, Li G, Quinn DG, et al. (1996) A point mutation in HLA-A*0201 results in failure to bind the TAP complex and to present virus-derived peptides to CTL. Immunity 4: 505–514.

8. Suh WK, Derby MA, Cohen-Doyle MF, Schoenhals GJ, Früh K, et al. (1999) Interaction of murine MHC class I molecules with tapasin and TAP enhances peptide loading and involves the heavy chain alpha3 domain. J Immunol 162: 1530–1540.

9. Yu YY, Turnquist HR, Myers NB, Balendiran GK, Hansen TH, et al. (1999) An extensive region of an MHC class I alpha 2 domain loop influences interaction with the assembly complex. J Immunol 163: 4427–4433.

10. Harris MR, Lybarger L, Myers NB, Hilbert C, Solheim JC, et al. (2001) Interactions of HLA-B27 with the peptide loading complex as revealed by heavy chain mutations. Int Immunol 13: 1275–1282.

11. Gao GF, Tormo J, Gerth UC, Wyer JR, McMichael AJ, et al. (1997) Crystal structure of the complex between human CD8alpha(alpha) and HLA-A2. Nature 387: 630–634. doi:10.1038/42523.

12. Kern PS, Teng MK, Smolyar A, Liu JH, Liu J, et al. (1998) Structural basis of CD8 coreceptor function revealed by crystallographic analysis of a murine CD8alphaalpha ectodomain fragment in complex with H-2Kb. Immunity 9: 519–530.

13. Liu Y, Xiong Y, Naidenko OV, Liu J, Zhang R, et al. (2003) The crystal structure of a TL/CD8alphaalpha complex at 2.1 A resolution: implications for modulation of T cell activation and memory. Immunity 18: 205–215.

14. Wang R, Natarajan K, Margulies DH (2009) Structural basis of the CD8 alpha beta/MHC class I interaction: focused recognition orients CD8 beta to a T cell proximal position. J Immunol 183: 2554–2564. doi:10.4049/jimmunol.0901276.

15. Shi Y, Qi J, Iwamoto A, Gao GF (2011) Plasticity of human CD8αα binding to peptide-HLA-A*2402. Mol Immunol 48: 2198–2202. doi:10.1016/j.molimm.2011.05.009.

16. Tormo J, Natarajan K, Margulies DH, Mariuzza RA (1999) Crystal structure of a lectin-like natural killer cell receptor bound to its MHC class I ligand. Nature 402: 623–631. doi:10.1038/45170.

17. Deng L, Cho S, Malchiodi EL, Kerzic MC, Dam J, et al. (2008) Molecular architecture of the major histocompatibility complex class I-binding site of Ly49 natural killer cell receptors. J Biol Chem 283: 16840–16849. doi:10.1074/jbc.M801526200.

18. Willcox BE, Thomas LM, Bjorkman PJ (2003) Crystal structure of HLA-A2 bound to LIR-1, a host and viral major histocompatibility complex receptor. Nat Immunol 4: 913–919. doi:10.1038/ni961.

19. Shiroishi M, Kuroki K, Rasubala L, Tsumoto K, Kumagai I, et al. (2006) Structural basis for recognition of the nonclassical MHC molecule HLA-G by the leukocyte Ig-like receptor B2 (LILRB2/LIR2/ILT4/CD85d). Proc Natl Acad Sci USA 103: 16412–16417. doi:10.1073/pnas.0605228103.

20. Chiu NM, Chun T, Fay M, Mandal M, Wang CR (1999) The majority of H2-M3 is retained intracellularly in a peptide-receptive state and traffics to the cell surface in the presence of N-formylated peptides. J Exp Med 190: 423–434.

21. Fremont DH, Stura EA, Matsumura M, Peterson PA, Wilson IA (1995) Crystal structure of an H-2Kb-ovalbumin peptide complex reveals the interplay of primary and secondary anchor positions in the major histocompatibility complex binding groove. Proc Natl Acad Sci USA 92: 2479–2483.

22. Wang J, Li Y, Kinjo Y, Mac T-T, Gibson D, et al. (2010) Lipid binding orientation within CD1d affects recognition of Borrelia burgorferi antigens by NKT cells. Proc Natl Acad Sci USA 107: 1535–1540. doi:10.1073/pnas.0909479107.

23. Porgador A, Yewdell JW, Deng Y, Bennink JR, Germain RN (1997) Localization, quantitation, and in situ detection of specific peptide-MHC class I complexes using a monoclonal antibody. Immunity 6: 715–726.

24. Mareeva T, Lebedeva T, Anikeeva N, Manser T, Sykulev Y (2004) Antibody specific for the peptide.major histocompatibility complex. Is it T cell receptor-like? J Biol Chem 279: 44243–44249. doi:10.1074/jbc.M407021200.

25. Mareeva T, Martinez-Hackert E, Sykulev Y (2008) How a T cell receptor-like antibody recognizes major histocompatibility complex-bound peptide. J Biol Chem 283: 29053–29059. doi:10.1074/jbc.M804996200.

26. Davignon JL, Guimezanes A, Schmitt-Verhulst AM (1983) Clonal analysis of H-2Kb + TNP recognition by T cells with the use of H-2Kbm mutants and H-2Kb-specific monoclonal antibodies. J Immunol 131: 1073–1079.

27. Hua C, Langlet C, Buferne M, Schmitt-Verhulst AM (1985) Selective destruction by formaldehyde fixation of an H-2Kb serological determinant involving lysine 89 without loss of T-cell reactivity. Immunogenetics 21: 227–234.

28. Nathenson SG, Geliebter J, Pfaffenbach GM, Zeff RA (1986) Murine major histocompatibility complex class-I mutants: molecular analysis and structure-function implications. Annu Rev Immunol 4: 471–502. doi:10.1146/annurev.iy.04.040186.002351.

29. Pullen JK, Hunt HD, Horton RM, Pease LR (1989) The functional significance of two amino acid polymorphisms in the antigen-presenting domain of class I MHC molecules. Molecular dissection of Kbm3. J Immunol 143: 1674–1679.

30. Jones B, Janeway CA Jr (1981) Cooperative interaction of B lymphocytes with antigen-specific helper T lymphocytes is MHC restricted. Nature 292: 547–549.

31. Ajitkumar P, Geier SS, Kesari KV, Borriello F, Nakagawa M, et al. (1988) Evidence that multiple residues on both the alpha-helices of the class I MHC molecule are simultaneously recognized by the T cell receptor. Cell 54: 47–56.

32. Davignon JL, Guimezanes A, Schmitt-Verhulst AM (1983) Clonal analysis of H-2Kb + TNP recognition by T cells with the use of H-2Kbm mutants and H-2Kb-specific monoclonal antibodies. J Immunol 131: 1073–1079.

33. Loken MR, Stall AM (1982) Flow cytometry as an analytical and preparative tool in immunology. J Immunol Methods 50: R85–112.

34. Rock KL, Gramm C, Benacerraf B (1991) Low temperature and peptides favor the formation of class I heterodimers on RMA-S cells at the cell surface. Proc Natl Acad Sci USA 88: 4200–4204.

35. Kuhns ST, Pease LR (1998) A region of conformational variability outside the peptide-binding site of a class I MHC molecule. J Immunol 161: 6745–6750.

36. Mitaksov V, Fremont DH (2006) Structural definition of the H-2Kd peptide-binding motif. J Biol Chem 281: 10618–10625. doi:10.1074/jbc.M510511200.

37. Kellenberger C, Roussel A, Malissen B (2005) The H-2Kk MHC peptide-binding groove anchors the backbone of an octameric antigenic peptide in an unprecedented mode. J Immunol 175: 3819–3825.

38. Honda M, Wang R, Kong W-P, Kanekiyo M, Akahata W, et al. (2009) Different vaccine vectors delivering the same antigen elicit CD8+ T cell responses with distinct clonotype and epitope specificity. J Immunol 183: 2425–2434. doi:10.4049/jimmunol.0900581.

39. Balendiran GK, Solheim JC, Young AC, Hansen TH, Nathenson SG, et al. (1997) The three-dimensional structure of an H-2Ld-peptide complex explains the unique interaction of Ld with beta-2 microglobulin and peptide. Proc Natl Acad Sci USA 94: 6880–6885.

40. Chu F, Lou Z, Chen YW, Liu Y, Gao B, et al. (2007) First glimpse of the peptide presentation by rhesus macaque MHC class I: crystal structures of Mamu-A*01 complexed with two immunogenic SIV epitopes and insights into CTL escape. J Immunol 178: 944–952.

41. Aricescu AR, Lu W, Jones EY (2006) A time- and cost-efficient system for high-level protein production in mammalian cells. Acta Crystallogr D Biol Crystallogr 62: 1243–1250. doi:10.1107/S0907444906029799.

42. Bendtsen JD, Nielsen H, von Heijne G, Brunak S (2004) Improved prediction of signal peptides: SignalP 3.0. J Mol Biol 340: 783–795. doi:10.1016/j.jmb.2004.05.028.

43. Alexander-Brett JM, Fremont DH (2007) Dual GPCR and GAG mimicry by the M3 chemokine decoy receptor. J Exp Med 204: 3157–3172. doi:10.1084/jem.20071677.

44. Mierendorf RC, Morris BB, Hammer B, Novy RE (1998) Expression and Purification of Recombinant Proteins Using the pET System. Methods Mol Med 13: 257–292. doi:10.1385/0-89603-485-2:257.

45. Studier FW (2005) Protein production by auto-induction in high density shaking cultures. Protein Expr Purif 41: 207–234.

46. Garboczi DN, Hung DT, Wiley DC (1992) HLA-A2-peptide complexes: refolding and crystallization of molecules expressed in Escherichia coli and complexed with single antigenic peptides. Proc Natl Acad Sci USA 89: 3429–3433.

47. Cohen SL, Ferré-D’Amaré AR, Burley SK, Chait BT (1995) Probing the solution structure of the DNA-binding protein Max by a combination of proteolysis and mass spectrometry. Protein Sci 4: 1088–1099. doi:10.1002/pro.5560040607.

48. Mitaksov V, Truscott SM, Lybarger L, Connolly JM, Hansen TH, et al. (2007) Structural engineering of pMHC reagents for T cell vaccines and diagnostics. Chem Biol 14: 909–922. doi:10.1016/j.chembiol.2007.07.010.

49. Walter TS, Meier C, Assenberg R, Au K-F, Ren J, et al. (2006) Lysine methylation as a routine rescue strategy for protein crystallization. Structure 14: 1617–1622. doi:10.1016/j.str.2006.09.005.

50. Vetting MW, Hegde SS, Blanchard JS (2009) Crystallization of a pentapeptide-repeat protein by reductive cyclic pentylation of free amines with glutaraldehyde. Acta Crystallogr D Biol Crystallogr 65: 462–469. doi:10.1107/S0907444909008324.

51. Dong A, Xu X, Edwards AM, Chang C, Chruszcz M, et al. (2007) In situ proteolysis for protein crystallization and structure determination. Nat Methods 4: 1019–1021. doi:10.1038/nmeth1118.

52. Heras B, Martin JL (2005) Post-crystallization treatments for improving diffraction quality of protein crystals. Acta Crystallogr D Biol Crystallogr 61: 1173–1180. doi:10.1107/S0907444905019451.

53. Otwinowski Z, Minor W (1997) [20] Processing of X-ray diffraction data collected in oscillation mode. Macromolecular Crystallography Part A. Academic Press, Vol. Volume 276. pp. 307–326. Available:http://www.sciencedirect.com/science/article/pii/S007668799776066X. Accessed 30 December 2011.

54. Long F, Vagin AA, Young P, Murshudov GN (2008) BALBES: a molecular-replacement pipeline. Acta Crystallogr D Biol Crystallogr 64: 125–132. doi:10.1107/S0907444907050172.

55. Auphan-Anezin N, Mazza C, Guimezanes A, Barrett-Wilt GA, Montero-Julian F, et al. (2006) Distinct orientation of the alloreactive monoclonal CD8 T cell activation program by three different peptide/MHC complexes. Eur J Immunol 36: 1856–1866. doi:10.1002/eji.200635895.

56. Adams PD, Afonine PV, Bunkóczi G, Chen VB, Echols N, et al. (2011) The Phenix software for automated determination of macromolecular structures. Methods (San Diego, Calif). Available:http://www.ncbi.nlm.nih.gov/pubmed/21821126. Accessed 30 September 2011.

57. Kelley LA, Sternberg MJE (2009) Protein structure prediction on the Web: a case study using the Phyre server. Nat Protoc 4: 363–371. doi:10.1038/nprot.2009.2.

58. Roy A, Kucukural A, Zhang Y (2010) I-TASSER: a unified platform for automated protein structure and function prediction. Nat Protoc 5: 725–738. doi:10.1038/nprot.2010.5.

59. Vonrhein C, Blanc E, Roversi P, Bricogne G (2007) Automated structure solution with autoSHARP. Methods Mol Biol 364: 215–230. doi:10.1385/1-59745-266-1:215.

60. Li W, Li F (2011) Cross-crystal averaging with search models to improve molecular replacement phases. Structure 19: 155–161. doi:10.1016/j.str.2010.12.007.

61. Cowtan K (1994) Joint CCP4 and ESF-EACBM Newsletter on Protein Crystallography. 31: 34–38.

62. Emsley P, Cowtan K (2004) Coot: model-building tools for molecular graphics. Acta Crystallogr D Biol Crystallogr 60: 2126–2132. doi:10.1107/S0907444904019158.

63. Kern PS, Teng MK, Smolyar A, Liu JH, Liu J, et al. (1998) Structural basis of CD8 coreceptor function revealed by crystallographic analysis of a murine CD8alphaalpha ectodomain fragment in complex with H-2Kb. Immunity 9: 519–530.

64. Reiser JB, Darnault C, Guimezanes A, Grégoire C, Mosser T, et al. (2000) Crystal structure of a T cell receptor bound to an allogeneic MHC molecule. Nat Immunol 1: 291–297. doi:10.1038/79728.

65. Mazza C, Auphan-Anezin N, Gregoire C, Guimezanes A, Kellenberger C, et al. (2007) How much can a T-cell antigen receptor adapt to structurally distinct antigenic peptides? EMBO J 26: 1972–1983. doi:10.1038/sj.emboj.7601605.

66. Deng L, Cho S, Malchiodi EL, Kerzic MC, Dam J, et al. (2008) Molecular architecture of the major histocompatibility complex class I-binding site of Ly49 natural killer cell receptors. J Biol Chem 283: 16840–16849. doi:10.1074/jbc.M801526200.

67. Mareeva T, Martinez-Hackert E, Sykulev Y (2008) How a T cell receptor-like antibody recognizes major histocompatibility complex-bound peptide. J Biol Chem 283: 29053–29059. doi:10.1074/jbc.M804996200.

68. Krissinel E, Henrick K (2004) Secondary-structure matching (SSM), a new tool for fast protein structure alignment in three dimensions. Acta Crystallogr D Biol Crystallogr 60: 2256–2268. doi:10.1107/S0907444904026460.

69. Kleywegt GJ, Read RJ (1997) Not your average density. Structure 5: 1557–1569.

70. Bränd´en C-I, Jones TA (1990) Between objectivity and subjectivity. Nature: 687 – 689.

71. Laskowski RA, MacArthur MW, Moss DS, Thornton JM (n.d.) PROCHECK - a program to check the stereochemical quality of protein structures. J App Cryst 26: 283–291.

72. Holm L, Rosenström P (2010) Dali server: conservation mapping in 3D. Nucleic Acids Res 38: W545–549. doi:10.1093/nar/gkq366.

73. Hayward S, Kitao A, Berendsen HJ (1997) Model-free methods of analyzing domain motions in proteins from simulation: a comparison of normal mode analysis and molecular dynamics simulation of lysozyme. Proteins 27: 425–437.

74. Madden DR (1995) The three-dimensional structure of peptide-MHC complexes. Annu Rev Immunol 13: 587–622. doi:10.1146/annurev.iy.13.040195.003103.

75. McDonald IK, Thornton JM (1994) Satisfying hydrogen bonding potential in proteins. J Mol Biol 238: 777–793. doi:10.1006/jmbi.1994.1334.

76. Krissinel E (n.d.) NCONT. Cambridge, UK: European Bioinformatics Institute. p.

77. Hubbard SJ, Thornton JM (1993) NACCESS. Department of Biochemistry and Molecular Biology, University College London. p.

78. Baker NA, Sept D, Joseph S, Holst MJ, McCammon JA (2001) Electrostatics of nanosystems: application to microtubules and the ribosome. Proc Natl Acad Sci USA 98: 10037–10041. doi:10.1073/pnas.181342398.

79. Larkin MA, Blackshields G, Brown NP, Chenna R, McGettigan PA, et al. (2007) Clustal W and Clustal X version 2.0. Bioinformatics 23: 2947–2948. doi:10.1093/bioinformatics/btm404.

80. Valdar WSJ (2002) Scoring residue conservation. Proteins 48: 227–241. doi:10.1002/prot.10146.

81. Achour A, Michaëlsson J, Harris RA, Ljunggren H-G, Kärre K, et al. (2006) Structural basis of the differential stability and receptor specificity of H-2Db in complex with murine versus human beta2-microglobulin. J Mol Biol 356: 382–396. doi:10.1016/j.jmb.2005.11.068.

82. Gewurz BE, Wang EW, Tortorella D, Schust DJ, Ploegh HL (2001) Human cytomegalovirus US2 endoplasmic reticulum-lumenal domain dictates association with major histocompatibility complex class I in a locus-specific manner. J Virol 75: 5197–5204. doi:10.1128/JVI.75.11.5197-5204.2001.

83. Moody AM, Xiong Y, Chang HC, Reinherz EL (2001) The CD8alphabeta co-receptor on double-positive thymocytes binds with differing affinities to the products of distinct class I MHC loci. Eur J Immunol 31: 2791–2799.

84. Natarajan K, Boyd LF, Schuck P, Yokoyama WM, Eliat D, et al. (1999) Interaction of the NK cell inhibitory receptor Ly49A with H-2Dd: identification of a site distinct from the TCR site. Immunity 11: 591–601.

85. Dam J, Guan R, Natarajan K, Dimasi N, Chlewicki LK, et al. (2003) Variable MHC class I engagement by Ly49 natural killer cell receptors demonstrated by the crystal structure of Ly49C bound to H-2K(b). Nat Immunol 4: 1213–1222. doi:10.1038/ni1006.

86. Kuroki K, Tsuchiya N, Shiroishi M, Rasubala L, Yamashita Y, et al. (2005) Extensive polymorphisms of LILRB1 (ILT2, LIR1) and their association with HLA-DRB1 shared epitope negative rheumatoid arthritis. Hum Mol Genet 14: 2469–2480. doi:10.1093/hmg/ddi247.

87. Chapman TL, Heikema AP, Bjorkman PJ (1999) The Inhibitory Receptor LIR-1 Uses a Common Binding Interaction to Recognize Class I MHC Molecules and the Viral Homolog UL18. Immunity 11: 603–613. doi:10.1016/S1074-7613(00)80135-1.

88. Bond CS, Schüttelkopf AW (2009) ALINE: a WYSIWYG protein-sequence alignment editor for publication-quality alignments. Acta Crystallogr D Biol Crystallogr 65: 510–512. doi:10.1107/S0907444909007835.

89. Bond CS (2003) TopDraw: a sketchpad for protein structure topology cartoons. Bioinformatics 19: 311–312.

90. Myszka DG (1999) Improving biosensor analysis. J Mol Recognit 12: 279–284. doi:10.1002/(SICI)1099-1352(199909/10)12:5<279::AID-JMR473>3.0.CO;2-3.

91. Abdiche Y, Malashock D, Pinkerton A, Pons J (2008) Determining kinetics and affinities of protein interactions using a parallel real-time label-free biosensor, the Octet. Anal Biochem 377: 209–217. doi:10.1016/j.ab.2008.03.035.

92. Kim JH, Johannes L, Goud B, Antony C, Lingwood CA, et al. (1998) Noninvasive measurement of the pH of the endoplasmic reticulum at rest and during calcium release. Proc Natl Acad Sci USA 95: 2997–3002.

93. Llopis J, McCaffery JM, Miyawaki A, Farquhar MG, Tsien RY (1998) Measurement of cytosolic, mitochondrial, and Golgi pH in single living cells with green fluorescent proteins. Proc Natl Acad Sci USA 95: 6803–6808.

94. Wu MM, Llopis J, Adams S, McCaffery JM, Kulomaa MS, et al. (2000) Organelle pH studies using targeted avidin and fluorescein-biotin. Chem Biol 7: 197–209.

95. Wu MM, Grabe M, Adams S, Tsien RY, Moore HP, et al. (2001) Mechanisms of pH regulation in the regulated secretory pathway. J Biol Chem 276: 33027–33035. doi:10.1074/jbc.M103917200.

96. Astafieva IV, Eberlein GA, Wang YJ (1996) Absolute on-line molecular mass analysis of basic fibroblast growth factor and its multimers by reversed-phase liquid chromatography with multi-angle laser light scattering detection. J Chromatogr A 740: 215–229.

97. Alexander JM, Nelson CA, van Berkel V, Lau EK, Studts JM, et al. (2002) Structural basis of chemokine sequestration by a herpesvirus decoy receptor. Cell 111: 343–356.

98. Greenfield NJ (2006) Using circular dichroism collected as a function of temperature to determine the thermodynamics of protein unfolding and binding interactions. Nat Protoc 1: 2527–2535. doi:10.1038/nprot.2006.204.
